# Selenium Nanoparticle-Enriched and Potential Probiotic, *Lactiplantibacillus plantarum* S14 Strain, a Diet Supplement Beneficial for Rainbow Trout

**DOI:** 10.3390/biology11101523

**Published:** 2022-10-18

**Authors:** Francisco Yanez-Lemus, Rubén Moraga, Carlos T. Smith, Paulina Aguayo, Kimberly Sánchez-Alonzo, Apolinaria García-Cancino, Ariel Valenzuela, Víctor L. Campos

**Affiliations:** 1 Environmental Microbiology Laboratory, Department of Microbiology, Faculty of Biological Sciences, Universidad de Concepcion, Concepcion 4070386, Chile; 2 Escuela de Medicina Veterinaria, Facultad de Recursos Naturales y Medicina Veterinaria, Universidad Santo Tomás, Santiago 8370003, Chile; 3 Microbiology Laboratory, Faculty of Renewable Natural Resources, Arturo Prat University, Iquique 1100000, Chile; 4 Faculty of Environmental Sciences, EULA-Chile, Universidad de Concepcion, Concepcion 4070386, Chile; 5 Institute of Natural Resources, Faculty of Veterinary Medicine and Agronomy, Universidad de Las Américas, Sede Concepcion, Chacabuco 539, Concepcion 3349001, Chile; 6 Laboratory of Bacterial Pathogenicity, Department of Microbiology, Faculty of Biological Sciences, Universidad de Concepcion, Concepcion 4070386, Chile; 7 School of Medical Technology, Faculty of Medicine and Science, Universidad San Sebastian, Concepcion 4080871, Chile; 8 Laboratory of Pisciculture and Aquatic Pathology, Department of Oceanography, Faculty of Natural and Oceanographic Sciences, Universidad de Concepcion, Concepcion 4070386, Chile

**Keywords:** nutritional supplement, probiotic, selenium nanoparticles, rainbow trout, *Oncorhynchus mykiss*, ROS, lysozyme, oxidative stress, production parameters

## Abstract

**Simple Summary:**

Potential probiotic bacteria for aquacultured species should be naturally occurring and non-pathogenic in the native habitat of the host, easy to culture, and able to grow in the intestine of the host. Se nanoparticles (Se^0^Nps) can be effectively used as a growth promoter, antioxidant, and immunostimulant agent in aquacultured species. Dietary supplementation with probiotics and Se^0^Nps contributes to the balance of the intestinal microbiota and probiotics have been proposed as an alternative to chemotherapeutants and antibiotics to prevent disease outbreaks, to mitigate the negative effects of stress and to strengthen the antioxidant capacity and the immune system of fish. Our results reported the isolation of a probiotic strain obtained from healthy rainbow trout. The strain was identified as *Lactiplantibacillus plantarum* species. This strain showed characteristics typically present in probiotics and, concurrently, the capacity to biosynthesize Se^0^Nps. The supplementation of the rainbow trout fish diet with LABS14-Se^0^Nps showed a positive effect on innate immune response parameters, oxidative status, well-being, and a better growth performance than the supplementation of the diet with the bacterium LABS14 alone. Therefore, we propose LABS14-Se^0^Nps as a promising alternative for the nutritional supplementation for rainbow trout or even other salmonids.

**Abstract:**

Lactic acid bacteria (LAB), obtained from rainbow trout (*Oncorhynchus mykiss*) intestine, were cultured in MRS medium and probiotic candidates. Concurrently, producers of elemental selenium nanoparticles (Se^0^Nps) were selected. Probiotic candidates were subjected to morphological characterization and the following tests: antibacterial activity, antibiotic susceptibility, hemolytic activity, catalase, hydrophobicity, viability at low pH, and tolerance to bile salts. Two LAB strains (S4 and S14) satisfied the characteristics of potential probiotics, but only strain S14 reduced selenite to biosynthesize Se^0^Nps. S14 strain was identified, by 16S rDNA analysis, as *Lactiplantibacillus plantarum*. Electron microscopy showed Se^0^Nps on the surface of S14 cells. Rainbow trout diet was supplemented (10^8^ CFU g^−1^ feed) with Se^0^Nps-enriched *L. plantarum* S14 (LABS14-Se^0^Nps) or *L. plantarum* S14 alone (LABS14) for 30 days. At days 0, 15, and 30, samples (blood, liver, and dorsal muscle) were obtained from both groups, plus controls lacking diet supplementation. Fish receiving LABS14-Se^0^Nps for 30 days improved respiratory burst and plasmatic lysozyme, (innate immune response) and glutathione peroxidase (GPX) (oxidative status) activities and productive parameters when compared to controls. The same parameters also improved when compared to fish receiving LABS14, but significant only for plasmatic and muscle GPX. Therefore, Se^0^Nps-enriched *L. plantarum* S14 may be a promising alternative for rainbow trout nutritional supplementation.

## 1. Introduction

Chile is a worldwide important producer of trout and salmon. In fact, it is the second largest aquacultured Atlantic salmon (*Salmo salar*) producer after Norway [1] and the leading rainbow trout (*Oncorhynchus mykiss*) producer in the world [2]. This high productivity requires intensive farming, i.e., massive fish biomass grown at high densities per unit of water volume, increasing the susceptibility of fish to diseases caused by various microbial pathogens, including bacteria [3]. Controlling bacterial fish diseases has been associated to an increased use of antibiotics and chemotherapeutics, leading to drug resistant pathogens. According to the official 2017 Aquaculture Environmental Report [4], the production of salmonids has caused the accumulation of organic matter (e.g., uneaten food, fish’s feces) and antibiotics in the sediment of sea, fjords, or lakes located directly beneath the fish cages. Urbina [5] reported a localized eutrophication and changes in overall microbial biodiversity in the sediments of different salmon culture centers in southern Chile. Cabello and Godfrey [6] suggested that the excessive use of antibacterials in the Chilean salmon aquaculture industry and the presence of antibacterials residues in the environment are creating a critical condition, inducing and spreading new antibacterial resistance genes in the bacterial communities with potentially negative effects on fish farming and human health.

The main antibiotics used in aquacultured salmonid fish are florfenicol and oxytetracycline. The Chilean governmental National Service of Fishing and Aquaculture (Sernapesca), during the 2017–2020 period, indicated that oxytetracycline and florfenicol represented 16.63% and 79.62%, respectively, of the total use of antibiotics in Chilean aquaculture [7]. Navarrete et al. [8] showed that oxytetracycline reduces bacterial diversity in the salmonid microbiota favoring opportunistic pathogenic bacteria proliferation. Donati et al. [9] reported that after 10 days of florfenicol dietary treatment, rainbow trout underwent a shift in the relative abundance, at the phylum level, of their intestinal microbiome, including an increase of *Proteobacteria* and a reduction of *Firmicutes* when compared to the control. Previously, Valdes et al. [10] had already demonstrated by metagenomic analysis that rainbow trout with an intestinal dysbiosis, with an increase of members of the *Proteobacteria* phylum and a reduction of members of the *Firmicutes* phylum, showed a higher susceptibility to flavobacteriosis, a freshwater disease caused by the Gram-negative bacterium *Flavobacterium psychrophilum.* Therefore, alternatives to reduce the use of these antibiotics are necessary.

The role of the intestinal microbiota in fish seems to be similar to that of terrestrial mammals, i.e., it reinforces the digestive and immune systems [11], promotes growth performance, and alleviates oxidative stress (OS) caused by toxic pollutants, such as the heavy metal cadmium (Cd) [12]. In fish, the imbalance of the intestinal microbiota is one of the most relevant consequences of the misuse of antibacterials. This imbalance may lead to the colonization or the overgrowth of opportunistic pathogenic bacteria, increasing fish mortality [13].

A number of alternatives of antibiotics in salmonid farming are available (Lozano et al., 2017). Among these alternatives, probiotics, “live microorganisms which when administered in adequate amounts confer a health benefit on the host” [14], improve nutrition, provide health benefits, reduce the prevalence of diseases, improve growth, health status, immunity, food conversion, microbial balance, and environmental-friendly food production [15,16]. The supplementation of food with probiotics may control various bacterial pathogens in several fish species [17], including rainbow trout [15]. The lactic acid produced by probiotics causes beneficial effects on a number of aquacultured species [18,19,20].

Lactic acid bacteria (LAB) are non-spore forming Gram-positive cocci or coccobacilli, including anaerobe or facultative anaerobe rods which produce organic acids, such as lactic acid, the main fermentation product of the metabolism of carbohydrate [21]. Some LAB bacterial strains, capable to produce bioactive compounds (such as lactic acid, acetate, formic acid, hydrogen peroxide, ethanol, enzymes, benzoate, antibacterial peptides, free fatty acids, and volatile compounds), can work synergistically as broad-spectrum antibacterials toward several pathogens and exert a probiotic activity [22].

Members of bacterial genera investigated as probiotics for salmonids include *Carnobacterium*, *Pediococcus* and *Lactobacillus,* which are LAB belonging to phylum *Firmicutes* [23]. Certain strains of *Lactiplantibacillus plantarum* (formerly *Lactobacillus plantarum*) have demonstrated probiotic properties [24] which have boosted the immune status and growth when supplemented to the diet in different fish species, such as the Atlantic salmon [25] and the rainbow trout [26,27].

On the other hand, Selenium (Se) is a chemical element indispensable for animals. Se is required in metabolic processes involved in development, growth, health, and fertility. It is a diet supplement required by cultured salmon and trout as a nutritional supplement [28,29]. Moreover, seleno-proteins, proteins requiring Se as cofactor, are involved in the removal of reactive oxygen species (ROS), preventing OS [30]. According to Rathore et al. [31], the elemental Se nanoparticles (Se^0^Nps) can be effectively used as a growth promoter, antioxidant, and immunostimulant agent in aquacultured species. Numerous other studies also reported the benefit of including Se^0^Nps in the diet of aquatic animals to enhance their growth performance, and their physiological and health condition [32,33,34]. The toxicity of Se^0^Nps is low toxicity and their functionality is high [35]. Markedly, when compared to other forms of Se, the inclusion of Se^0^Nps as a food supplement has proven to better enhance growth performance and productivity in aquatic animals [36].

Bacteria are microorganisms characterized by their capacity to grow rapidly, being easy to handle and the cost to culture them is relatively low. Therefore, they are micro-factories able biosynthesize, among other compounds, metal nanoparticles [37]. Besides their easy processing, the low environmental impact, and the pharmacological merits, the production of Se^0^Nps by bacteria has become an extensively validated method [38]. A number of LAB have been investigated as Se-enriched (i.e., bacteria capable to produce Se^0^Nps) food supplement applications [39]. Some strains of *L. plantarum* have demonstrated to be able to accumulate Se salts and to biotransform amino acids into seleno-amino acids or Se^0^Nps [40,41,42]. A Se-enriched *L. plantarum* supplemented diet has been shown to protect against Cd toxicity, reducing OS in the fish *Luciobarbus capito* [43] and having anti-inflammatory and immunomodulatory effects in mice [44].

Considering the benefits that probiotics on one hand and Se^0^NPs on the other hand can provide to the salmonid farming industry, this study aimed firstly to isolate and select, from the intestinal content of rainbow trout, a suitable lactic acid bacterial strain possessing the characteristics of a probiotic and concurrently being able to produce Se^0^Nps and then to evaluate, in vivo, its possible positive effect, when administered as a diet supplement, on innate immune response, the oxidative status, and productive parameters of rainbow trout.

## 2. Materials and Method

### 2.1. Animals Used

All rainbow trout (*O. mykiss*) subjected to the following assays were treated according to the Biosecurity Regulations and Ethical Protocols approved by the University of Concepcion (UdeC) Ethics Committee (protocol code CBB 1084-2021). A total of 108 apparently healthy rainbow trout were used in this study. All fish were obtained from a fish farm (Florida, BioBio Region, Chile) and transported to the UdeC facilities (approximately 25 km distance) considering the guidelines for the welfare of farmed fish during transport included in the Aquatic Animal Health Code [45].

The plan to be accomplished in the present study firstly included to isolate putative LAB from the intestinal content of 6 fish to search for isolates showing characteristics of potential probiotics as well as the ability to produce Se^0^Nps. Then, considering its probiotic potential and Se^0^Nps production, a selected isolate was to be selected to be dispensed, as a food supplement, to rainbow trout to evaluate, in vivo, its effect on the innate immune response, on the oxidative status, and on the productive parameters of *O. mykiss*. The in vivo work plan required a total of 102 fish (96 fish required for the in vivo trials plus 6 additional spare fish).

### 2.2. Obtention of Putative LAB Isolates from the Intestinal Content of Rainbow Trout

Six fish (average weight 105.7 ± 3.2 g) were transferred to the Laboratory of Environmental Microbiology (LEM), UdeC, where they were euthanized using an overdose (50 ppm) of BZ-20 (sodium para-aminobenzoate) anesthetic (Veterquimica, Santiago, Chile) following indications given by the American Veterinary Medical Association (AVMA) guidelines for euthanizing animals [46]. Then, their intestines were aseptically removed, with the intestinal content of the 6 fish mixed and homogenized. Subsequently, 1 g of this homogenate was suspended in 9 mL sterile saline solution, vigorously vortexed by at least 2 min, and then transferred to a 15 mL Falcon tube (Corning Inc., Tewksbury, MA, USA). Then, 100 µL of serial dilutions (10^−1^ to 10^−7^) were transferred to plates containing Man, Rogosa and Sharpe (MRS) agar (Merck, Darmstadt, Germany), a culture medium specially designed to allow the growth of most LAB strains [47], and then incubated under microaerobic condition at 37 °C for 48 h using the candle jar method [48]. For the identification of colonies as LAB, the macroscopic morphological characterization of colonies was analyzed, and the Gram stain and catalase test were used. Gram-positive, catalase negative colonies were chosen for carbohydrate fermentation tests. Each colony that showed a carbohydrate fermentative metabolism was considered a LAB isolate [49,50]. The experiments were carried out in triplicate.

### 2.3. Search for Isolates from the Intestinal Content of Rainbow Trout Having Characteristics of LAB

#### 2.3.1. Morphological Characterization of Isolates

The morphology of each colony (hereafter referred as isolate) was evaluated visually in pure cultures in Petri dishes as described by Procop et al. [51]. These observations included the shape, color, edges, and elevation of the colonies. The observation of bacterial cells was performed, after Gram staining, under a light microscope (Olympus CX31, Tokyo, Japan), in order to select the Gram-positive isolates [52].

#### 2.3.2. Catalase Test

The catalase test was performed by placing two drops of 3% hydrogen peroxide on an object glass slide in which each isolate from a 24–48 h culture at 37 °C under microaerobic conditions was previously spread. A catalase test was considered as positive when bubbles, resulting from the activity of the bacterial catalase enzyme which converts H_2_O_2_ into water and oxygen, were observed [53]. The experiments were carried out in triplicate.

#### 2.3.3. Carbohydrate Fermentation Tests

Carbohydrate fermentation tests were performed according to Erkus [54] with modifications. Briefly, 6 sugar substrates, 3 hexoses (glucose, fructose, and galactose) and 3 pentoses (ribose, xylose and arabinose) (Merck, Darmstadt, Germany), were used. Sugars were independently dissolved in deionized water at final concentrations of 5% (*w*/*v*), and each solution was sterilized using 0.22 μm pore diameter sterile syringe filters (Thermo Fisher Scientific, Göteborg, Sweden). MRS broth plus 0.01 g phenol red (Merck, Darmstadt, Germany) per L of broth, as pH indicator, was prepared. Then, 4.5 mL of MRS broth plus phenol red were placed into screw cap test tubes and, after placing Durham’s tubes, they were autoclaved at 121 °C for 15 min. Each sugar sterile solution (0.5 mL) was added to different test tubes and 200 µL of each isolate (previously adjusted to 0.5 McFarland) were inoculated into the MRS broth containing phenol red. Incubation was performed under microaerobic condition at 37 °C for 24–48 h. Carbohydrate fermentation was detected by the color change of the medium and gas formation was detected in the Durham tubes [54].

### 2.4. Search for Potential Probiotic Characteristics in the Isolated LAB Strains

The isolates classified as LAB strains were tested to determine, according to Rondón et al. [55], if they possessed characteristics of potential probiotics. Their antibacterial activity, antibiotic susceptibility, viability at a low pH, tolerance to bile salts, hemolytic activity, and hydrophobicity of isolates were evaluated using assays. The experiments were carried out in triplicate.

#### 2.4.1. Antibacterial Activity of the LAB Strains

Isolates were individually screened to detect their antibacterial activity against indicator bacteria. Indicator bacteria included both Gram-positive (*Staphylococcus aureus* ATCC 25923 and *Bacillus subtilis* ATCC 6633 strains) and Gram-negative bacteria (*Escherichia coli* ATCC 25922 and *Pseudomonas aeruginosa* ATCC 10145 strains) and were tested according to Schillinger & Lücke [56] and Geria et al. [57] with modifications. Briefly, each LAB strain, adjusted to 0.5 McFarland, was individually sown by swabbing on Petri dishes containing a thin layer of MRS agar and then 4 mm in diameter discs were aseptically removed. One disc of each LAB strain was placed on top of trypticase soy agar (TSA) (Merck, Darmstadt, Germany) containing Petri dishes in which the indicator bacteria (adjusted to 0.5 McFarland) had been previously sown. Finally, the Petri dishes were incubated at 37 °C for 24–48 h under microaerobic conditions. Diameters of inhibition halos observed were recorded. The absence of inhibition halo was interpreted as negative antibacterial activity [58]. LAB strains and indicator bacteria were also separately cultured under similar conditions as growth controls. The experiments were carried out in triplicate.

#### 2.4.2. Antibiotic Susceptibility Test of the LAB Strains

The phenotypic susceptibility of LAB strains to antibiotics was determined by means of the agar diffusion method as indicated by the Clinical and Laboratory Standards Institute (CLSI) [59]. Susceptibility to: gentamicin (GEN; 10 µg), tetracycline (TET; 30 µg), oxytetracycline (OXY; 30 µg), erythromycin (ERY; 15 µg), florfenicol (FLO; 30 µg), and ampicillin (AMP; 10 µg) (Oxoid, Hampshire, United Kingdom) was tested. Briefly, each LAB strain was grown in MRS broth at 37 °C for 18 h under microaerobic conditions from which a 0.5 McFarland inoculum was prepared. Then, 100 µL of cell suspension were evenly spread on a Mueller-Hinton agar (Merck, Darmstadt, Germany) containing plate and maintained at room temperature for 1 h. Antibiotic discs were aseptically placed on the plates and the plates were incubated at 37 °C for 24–72 h under microaerobic condition. Antibacterial susceptibility was interpreted according to the inhibition diameter disc diffusion breakpoint proposed by the CLSI [59] (Table 1). The experiments were carried out in triplicate.

#### 2.4.3. Hemolytic Activity of the LAB Strains

To determine the hemolytic activity of the LAB strains, the method of Rodrigues et al. [61], with modifications, was used. Briefly, bacteria from an axenic and fresh culture were sown on MRS agar containing 5% human blood. The plates were then incubated at 37 °C for 24, 48, or 72 h under microaerobic conditions, after which alpha, beta or gamma hemolysis around each colony was determined. *S. aureus* ATCC 6538 was used as a positive control. The experiments were carried out in triplicate.

#### 2.4.4. Hydrophobicity Assays

To determine the hydrophobicity of the LAB strains, the MATH method by Xu et al. [62] was used with modifications. Briefly, 2 mL of a bacterial suspension of each LAB strain adjusted to 0.5 McFarland was combined with 0.8 mL p-xylene (1,4-dimethylbenzene; Merck, Darmstadt, Germany) and vortexed for 2 min. Samples were maintained at room temperature and the phases allowed to separate by decantation and the aqueous phase removed. The decrease in the absorbance of the aqueous phase, at an optical density (OD) of 600 nm, was considered a measure of cell surface hydrophobicity (H%), which was calculated using the formula:

$$H\%=\frac{A_{0}-A}{A_{0}}\times 100.\;$$

where *A*_0_ and *A* are the absorbances before and after extraction with p-xylene, respectively. The experiments were carried out in triplicate.

According to Sánchez-Ortiz et al. [63], H% values < 30% were considered as “Low”, values ≥ 30%, <60% were referred to as “Medium”, and values ≥ 60% were referred to as “High”. Strains with low adhesion to p-xylene (<30%) were discarded as potential probiotics.

#### 2.4.5. Cell Viability of the LAB Strains at a Low pH or Bile Salts

Tolerance of the selected LAB strains to acidic pH or bile salts was determined based on the methodology of Kaushik et al. [64] and Klayraung and Okonogi [53], respectively, with modifications. Briefly, 1 mL of bacterial culture was grown in 9 mL MRS broth adjusted to pH 3 using 5 N HCl (Merck, Darmstadt, Germany) or supplemented with 0.3% be salts (Ox-bile dehydrated and purified salt for microbiology, Merck, Darmstadt, Germany) [65] at 37 °C for 4 h under microaerobic conditions. Then, aliquots were transferred to plates containing MRS agar and incubated at 37 °C for 48 h under microaerobic conditions and counts expressed as log CFU mL^−1^. LAB strains cultured under similar conditions but not subjected to the low pH or bile salts were used as controls. The viability of LAB strains subjected to the acidic pH or to the bile salts as a percentage of viable cells with respect to the control was assessed according to the formula:

$$\%\;\mathrm{cell}\;\mathrm{viability}=\frac{\mathrm{Number}\;\mathrm{of}\;\mathrm{CFU}\;\mathrm{LAB}\;\mathrm{strain}\;\mathrm{in}\;\mathrm{MRS}\;\mathrm{exposed}\;\mathrm{low}\;\mathrm{pH}\;\mathrm{or}\;\mathrm{bile}\;\mathrm{salts}\;}{\mathrm{Number}\;\mathrm{of}\;\mathrm{CFU}\;\mathrm{LAB}\;\mathrm{strain}\;\mathrm{in}\;\mathrm{MRS}\;\mathrm{not}\;\mathrm{exposed}\;\mathrm{to}\;\mathrm{low}\;\mathrm{pH}\;\mathrm{or}\;\mathrm{bile}\;\mathrm{salts}}\times 100$$

A LAB strain was considered as tolerant to low pH or bile salts if counts of CFU mL^−1^ of the LAB strain cultured under a low pH or to bile salts was higher than 50% counts of CFU mL^−1^ of the respective control. The experiments were carried out in triplicate

### 2.5. Biosynthesis and Characterization of Se^0^Nps-Enriched Probiotic Strain (LABstrain-Se^0^Nps)

The LAB strains that showed the most promising characteristics of a potential probiotic bacterium according to Rondón et al. [55] were individually cultured in MRS agar containing 1 mM Na_2_SeO_3_ at 37 °C for 24 h under microaerobic condition [48]. Colonies which acquired a red color, a characteristic feature of the allotropic form of the Se^0^ [66], were transferred to 1.5 mL Eppendorf tubes (Merk, Darmstadt, Germany) containing 500 µL of previously sterilized distilled water and tubes centrifuged at 10,000× *g* for 10 min in a Universal 320|320 R centrifuge (Andreas Hettich GmbH & Co., KG, Tuttlingen, Germany). The supernatants of each tube were discarded, and the pellets washed three times using 500 µL sterile distilled water. After the last wash, distilled water was discarded and replaced by 500 µL 2.5% glutaraldehyde (Merck, Darmstadt, Germany) in cacodylate buffer (Merck, Darmstadt, Germany) [67]. Samples were processed at the Laboratory of Electron Microscopy (UdeC) for characterization by transmission electron microscopy (TEM) as described by Dhanjal and Cameotra [68] using a JEOL JSM 1200EX-II TEM microscope (JEOL, Peabody, MA, USA) and scanning electron microscopy (SEM) for their visualization and SEM-Energy Dispersive X-ray Spectroscopy (SEM-EDS) for their chemical characterization, as described by Torres et al. [69], using a JEOL JSM 6380LV SEM microscope (JEOL, Peabody, MA, USA). The experiments were carried out in triplicate.

### 2.6. DNA Isolation, 16S rDNA Gene Amplification and Sequencing of LAB Strain-Se^0^Nps

DNA was extracted from each LAB strain making use of the Dneasy UltraClean Microbial kit (Qiagen, Hilden, Germany), in accordance with the manufacturer’s indications. The bacterial DNA of every single isolate was amplified by PCR according to a method described by Wang et al. [70] using 16S rDNA universal primers GM3f (5′-AGAGTTTGATCMTGGC-3′) and GM4r (5′-TACCTTGTTACGACTT-3′) [71]). The products were sequenced by the method of Sanger’s using an ABI PRISM 3500 xL Genetic Analizer (Applied Biosystems, Foster City, CA, USA) [71]. The sequencing was done at Genoma Mayor (Universidad Mayor, Santiago, Chile). The sequences were analyzed using the Basic Local Alignment Search Tools (BLAST).

### 2.7. Effects of the LAB Strain-Se^0^Nps Dietary Supplementation on the Innate Immune Response, the Oxidative Status, and Productive Parameters of Rainbow Trout

#### 2.7.1. Rainbow Trout Rearing Conditions and Experimental Design

One hundred and two fish were transported to a semi-closed and environmental controlled recirculation system at the Laboratory of Pisciculture and Aquatic Pathology (LPAP), Faculty of Natural and Oceanographical Sciences (Universidad de Concepción). The 102 fish were kept in fiberglass tanks at a density of 25 kg fish m^−3^ under a 12:12 light:dark photoperiod [72]. The water quality parameters were monitored and recorded daily during the time fish were maintained at the LPAP (up to 51 days). The daily average of the parameters was, temperature: 14.9 ± 1.3 °C, dissolved oxygen: 8.3 ± 0.12 mg L^−1^, ammonia (total ammonia nitrogen ≤0.1 mg L^−1^), nitrite (≤0.2 mg L^−1^) and pH: 7.6 ± 0.7 (values ± correspond to the maximum daily variation recorded).

Rainbow trout were maintained during a 21-day acclimation period at the LPAP facilities before starting the assays. During adaptation, fish received a commercial extruded food acclimation diet (AD) (Cargill-Ewos, Coronel, Chile). The composition of the Cargill-Ewos commercial extruded food is reported in Table 2. Fish were feed two times per day, at 10:00 h and 16:00 h with 2% of their average body weight. At the beginning of the assay and on sampling days, eight randomly selected fish from each tank were weighed (BLC 1500 scale, Boeco, Hamburg, Germany) to adjust the amount of food supplied. After the adaptation period, fish were randomly distributed in 6 tanks (17 fish per tank, 2 tanks per diet) and there fed, for 30 days. Diet D1 corresponded to AD plus the LAB strain selected for having properties of a probiotic and producer of Se^0^Nps when cultured in the presence of Na_2_SeO_3_. Diet D2 was similar to D1 except that the selected bacterial strain was cultured in the absence of Na_2_SeO_3_ to avoid the presence of Se^0^Nps. The control diet was the same as AD.

#### 2.7.2. Preparation of Diets

The LAB strain selected to enrich D1 and D2 was cultured in MRS broth with or without 1 mM Na_2_SeO_3_, respectively, with agitation (100 rpm) in 2000 mL Erlenmeyer flasks (Merck, Darmstadt, Germany) at 37 °C for 24 h under microaerobic condition. Then, cultures were transferred to 50 mL conical Falcon centrifuge tubes and centrifuged at 10,000× *g* for 10 min in a Universal 320|320 R centrifuge. The supernatant of each tube was discarded, and the pellet was washed thrice using 500 µL sterile distilled water. After the last wash, distilled water was discarded and replaced by sterilized saline solution at a 1:4 (pellet:saline solution) ratio.

To prepare the live spray diets D1 and D2, 10^8^ CFU LAB strain per g of dry AD was used as indicated by Vera [73]. All diets were prepared weekly. Care was taken to maintain sterile conditions through all procedures. The stock diets were kept at 20 ± 2 °C. The viability of the LAB strain incorporated to the diets D1 and D2 was tested after vortexing 10 g of each diet in 90 mL of peptone water and spreading 0.1 mL aliquots of serial dilutions, in triplicate, on plates containing MRS agar and incubating them at 37 °C under microaerobic conditions for 48 h. Finally, the number of CFU was counted.

#### 2.7.3. Rainbow Trout Sampling

On days 0, 15, and 30, six fish from each group (it being the experimental or control group) were taken from the tanks at the LPAP and anesthetized by immersion in fresh water containing 50 ppm of BZ-20 (sodium para-aminobenzoate) (Veterquimica, Santiago, Chile) until stage III anesthesia in fish was observed (total loss of equilibrium and reactivity but opercular movement present) [74,75]. Then, rainbow trout were individually weighed using a BLC 1500 scale and measured (tip of the snout to the rear edge of the fork at the tail center). Subsequently, blood was withdrawn, using a heparinized 18G needle and a syringe, from the caudal vein of each fish and transferred to sterile microtubes containing 0.02 mL of 1000 U mL^−1^ heparin sodium salt (Merck, Darmstadt, Germany). Once blood was obtained, each sampled fish was humanely euthanized, as described in Section 2.2. Samples of the liver and of the dorsal muscle of each fish were obtained on day 30 of experimentation after the euthanasic procedure was concluded. Blood, liver, and dorsal muscle samples were promptly carried, at 4 °C, to the LEM, UdeC (distance approximately 400 m). Blood obtained from each fish was divided into 2 parts, one used to isolate white blood cells (WBC), as described by Hu et al. [76], and the other to obtain plasma by centrifugation at 5000× *g* for 10 min. Liver and dorsal muscle were fragmented. Then, plasma, liver, and dorsal muscle were stored at −80 °C. WBC were used to determine ROS concentration immediately after being obtained.

#### 2.7.4. Evaluation of ROS in White Blood Cells and Lysozyme Activity in Plasma

ROS concentration in WBC and plasmatic lysozyme activity of six fish were measured each sampling day per diet. The ROS assay evaluated the reduction, by oxidizing agents, of nitroblue tetrazolium (NBT) into spectrophotometrically measurable formazan [77]. Briefly, 100 μL of the WBC suspension in Ringer’s solution from each experimental or control fish containing 1 × 10^7^ WBC mL^−1^ were incubated, with 100 μL 0.1% NBT (Merck, Darmstadt, Germany) in tubes of 2 mL (Eppendorf, Hamburg, Germany) for 60 min. Posteriorly, 1 mL N,N-dimethylformamide (Merck, Darmstadt, Germany) was added and the tubes of 2 mL (Eppendorf, Hamburg, Germany), for 10 min at 100× *g*. The absorbance, measured at 620 nm, of the supernatant was determined using an Epoch spectrophotometer.

The plasmatic lysozyme activity was assessed using a turbidimetric assay [78]. Briefly, after plasma was thawed on ice, an aliquot of 50 µL was combined with 950 µL of a mixture containing 0.25 mg of *Micrococcus lysodeikticus* in 1 mL of buffered 40 mM sodium phosphate pH 6.2. The absorbance of this suspension was quantified at 450 nm wavelength by means of an Epoch spectrophotometer immediately after adding plasma (time 0) and after 30 min. A 0.001 min^−1^ absorbance reduction was valued as one unit of lysozyme activity [78]. ROS in WBC and plasmatic lysozyme activity measurements were carried out in triplicate.

#### 2.7.5. Activity of the Antioxidant Enzyme Glutathione Peroxidase (Gpx) in Plasma, Liver and Dorsal Muscle

The Gpx activity was measured in plasma following the indications of Lawrence and Burk [79] and in the liver and in the dorsal muscle those of Fontagné-Dicharry et al. [80]. For plasma, Gpx activity was evaluated immediately after thawing. For liver and muscle, after thawing the samples on ice and homogenizing them in 10 volumes (*w*/*v*) of ice-cold saline for 3 min, they were centrifuged for 15 min at 4000× *g* before the activity of GPx was determined in the supernatants. GPx activity was measured in a solution of 50 mM phosphate buffer (pH 7.4), 1 mM EDTA (Merck, Darmstadt, Germany), 2 mM sodium azide (Merck, Darmstadt, Germany), 2 mM reduced glutathione (GSH) (Merck, Darmstadt, Germany), 0.1 mM NADPH (Merck, Darmstadt, Germany), and 0.2 mM glutathione reductase (Merck, Darmstadt, Germany). H_2_O_2_ (50 μM) reduction at 30 °C was measured at 340 nm in an Epoch spectrophotometer. One unit of Gpx activity was valued as 1 mol NADPH consumed per min per mg of plasmatic proteins, using the appropriate molar absorptivity coefficient for NADPH (6220 mol L^−1^ cm^−1^). Plasmatic protein measurement was performed following the method of Lowry et al. [81].

#### 2.7.6. Effect of Diets on Trout Growth Performance and Survival Rate

On day 30, the effects of D1 or D2, in comparison to AD (control), on the productive parameters of the fish were evaluated. The weight and length of each trout and the number of dead fish were recorded to evaluate the specific growth rate (*SGR*), weight gain (*WG*), condition factor (*CF*), and survival percentage. The parameters were calculated as described by Naderi et al. [82] and Lugert et al. [83], using the formula:

$$SRG\;\left(\%\;increase\;body\;wt\;d^{-1}\right)=\left[\frac{\left(\ln w2-\ln w1\right)}{days}\right]\times 100$$

$$WG\;\left(g\right)=w2-w1$$

$$CF=\left[\frac{w}{L^{3}}\right]\times 100\;$$

$$Survival\;rate\;\left(\%\right)=\left[\frac{n2}{n1}\right]\times 100$$

where *w*1 = starting weight (g); *w*2 = final weight (g); *days* = days in the growth period; *w* = weight (g); *L* = length (cm); *n*1 = initial number of fish; *n*2 = final number of fish.

Fish of groups D1, D2, and AD were made up considering an initial condition factor (ICF) (similar sizes and weights) to ensure that the initial populations of the groups were homogeneous regarding their development stage and nutritional condition.

### 2.8. Statistics

One-way analysis of variance (ANOVA) associated to a power and sample size test followed by a Fisher’s least significant difference (LSD) multiple comparison test allowed to determine the statistical significance for multiple comparisons. The student’s *t*-test was used for pairwise comparisons. *p* < 0.05 values were considered as statistically significant. GraphPad Prism software version 7 for Windows (GraphPad Software, La Jolla, CA, USA) was used for all statistical tests executed.

## 3. Results

### 3.1. Isolation of LAB

Culturable bacteria from the intestinal content of six apparently healthy rainbow trout were obtained in MRS agar medium. After 12 h of incubation at 37 °C under microaerobic condition, colonies were visible in all agar plates. After 48 h of incubation, 16 colonies slightly white, convex, circular, with defined edges, 2–5 mm in diameter, and creamy consistency were observed. Each colony was collected and individually referred to as S1–S16 isolate. After light microscopy observations (Gram staining) and the catalase test were performed using the 16 isolates, six rod-shaped Gram-positive and catalase negative isolates were selected (S2, S4, S8, S12, S13, and S14) (Table 3) before they were subjected to the carbohydrate fermentation test by each isolate. The results are displayed in Table 4. From these findings, isolates S2, S4, S8, S12, S13, and S14 isolates were considered as LAB strains.

### 3.2. Evaluation of the Attributes of a Probiotic in the LAB Strains

#### 3.2.1. Antibacterial Activity of the LAB Strains

Except for the LAB strain S12 (LABS12), the rest of them (LABS2, LABS4, LABS8, LABS13, and LABS14) showed antibacterial activity against the indicator bacterial strains (*S. aureus* ATCC 25923, *B. subtilis* ATCC 6633, *E. coli* ATCC 25922 and *P. aeruginosa* ATCC 10145). The inhibition halos produced by the rainbow trout intestinal strains assayed on the indicator strains (Table 5) ranged from 8 mm (LABS8 against *E. coli*) to 31 mm (LABS4 against *P. aeruginosa*). Gram-positive indicator bacteria were shown to be more susceptible than Gram-negative to the antibacterial activity of the strains, being mostly and more significantly inhibited (*p* < 0.05) by LABS4 and LABS14strains than by strains LABS2, LABS8, and LABS13. Gram-negative reference bacterial strain *E. coli* ATCC 25922 was mainly inhibited by LABS2 (*p* < 0.05) followed by LABS4, both having a significantly higher activity than LABS8, LABS13, and LABS14. *P. aeruginosa* ATCC 10145 strain was mainly inhibited by LABS4, followed by LABS14 both having a significantly higher activity than LABS2, LABS8, and LABS13 strains (*p* < 0.05). Considering that LABS12 showed the poorest antibacterial activity against the indicator bacteria, it was not included in the following assays. LABS12 was unable to produce an inhibition halo against *S. aureus* ATCC 25923, *E. coli* ATCC 25922, and *P. aeruginosa* ATCC 10145.

#### 3.2.2. Antibiotic Susceptibility of the LAB Strains

**Figure 1:**
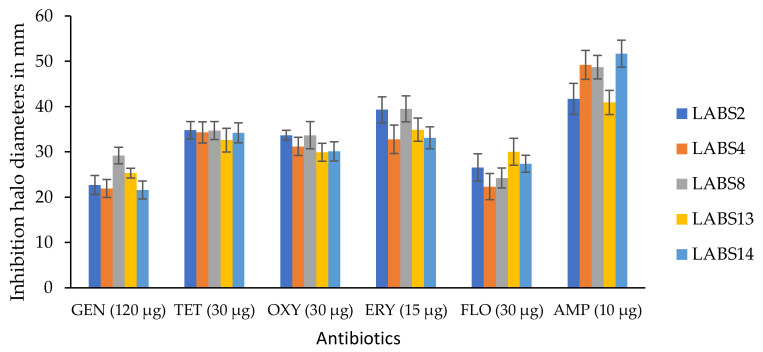
Antibiotic susceptibility of the 5 LAB strains (LABS2, LABS4, LABS8, LABS13 and LABS14), obtained from the intestinal content of rainbow trout to the antibacterial gentamycin (GEN), tetracycline (TET), oxytetracycline (OXY), erythromycin (ERY), florfenicol (FLO), and ampicillin (AMP), evaluated by the disc diffusion antibiotic method according to CLSI (2022) [59]. Data is given as mean ± SD. Experiments were carried out in triplicate.

The susceptibility of the five LAB strains remaining as part of this study (LABS2, LABS4, LABS8, LABS13 and LABS14) to the antibacterial GEN, TET, OXY, ERY, FLO, and AMP was determined by the disc diffusion method. The susceptibility shown by the five LAB strains is summarized in Figure 1. According to the criteria indicated in Table 1 (Section 2.4.2), all the isolates were demonstrated to be susceptible to every one of the antibiotics tested.

#### 3.2.3. Hemolytic Activity

The hemolytic activity of the five LAB strains (LABS2, LABS4, LABS8, LABS13, and LABS14) was evaluated in MRS agar plus 5% human blood after 24 h, 48 h, and 72 h of incubation (Table 6). Because gamma hemolysis is consistent with a LAB strain with probiotic properties, LABS4 and LABS14 were selected for the following assays.

#### 3.2.4. Hydrophobicity Assays

The MATH test, a method used to evaluate the ability of cells to adhere to the surface of another cell, was assessed for LABS4 and LABS14 measuring absorbance at 600 nm. LABS4 (30%) and LABS14 (37%) showed a medium hydrophobicity. Therefore, LABS4 and LABS14 were selected to evaluate their viability at a low pH.

#### 3.2.5. Cell Viability of the LAB Strains at Low pH

The tolerance at low pH, measured as viability of LABS4 and LABS14 at pH 3, is summarized in Table 7. LABS4 and LABS14 were tolerant to pH 3 showing a viability of 57.1% and 74.8%, respectively, when compared with their respective controls. LABS14 was significantly more resistant to pH 3.0 than LABS4 (*p* < 0.05). Even though the number of CFU recorded from LABS4 or LABS14 strains exposed to pH 3.0 was significantly reduced when compared to their respective controls (*p* < 0.05), they satisfied the criterium to consider them as tolerant to a low pH.

#### 3.2.6. Cell Viability of the LAB Strains in the Presence of Bile Salts

The tolerance to bile salts, measured as viability of LABS4 and LABS14 strains when subjected to 0.3% (*w*/*v*) bile salts, is summarized in the Table 6. LABS4 and LABS14 were tolerant to 0.3% bile salts with 69.2% and 82.3% viability, respectively, when compared with their respective controls. LABS14 was significantly more resistant to 0.3% bile salts than LABS4 (*p* < 0.05). Although the number of CFU recorded from LABS4 or LABS14 strains exposed to bile salts was significantly reduced when compared to respective controls (*p* < 0.05), the criterium to consider them as tolerant was fulfilled.

### 3.3. Screening for Biosynthesis of Se^0^Nps by LAB Strains (LABstrain-Se^0^Nps)

The ability of LABS4 or LABS14 to convert Na_2_SeO_3_ into Se^0^Nps was tested. The red color of colonies cultured in medium containing Na_2_SeO_3_ confirms the transformation of Na_2_SeO_3_ into Se^0^ and the possible capacity of LAB strains to produce Se^0^Nps. Only LABS14 colonies showed red color after being incubated at 37 °C for 24 h in MRS agar plus 1 mM Na_2_SeO_3_. LABS4 failed to produce a red color and was therefore excluded from the remaining experiments.

### 3.4. Characterization of Se Nanoparticles Produced by LABS14

**Figure 2:**
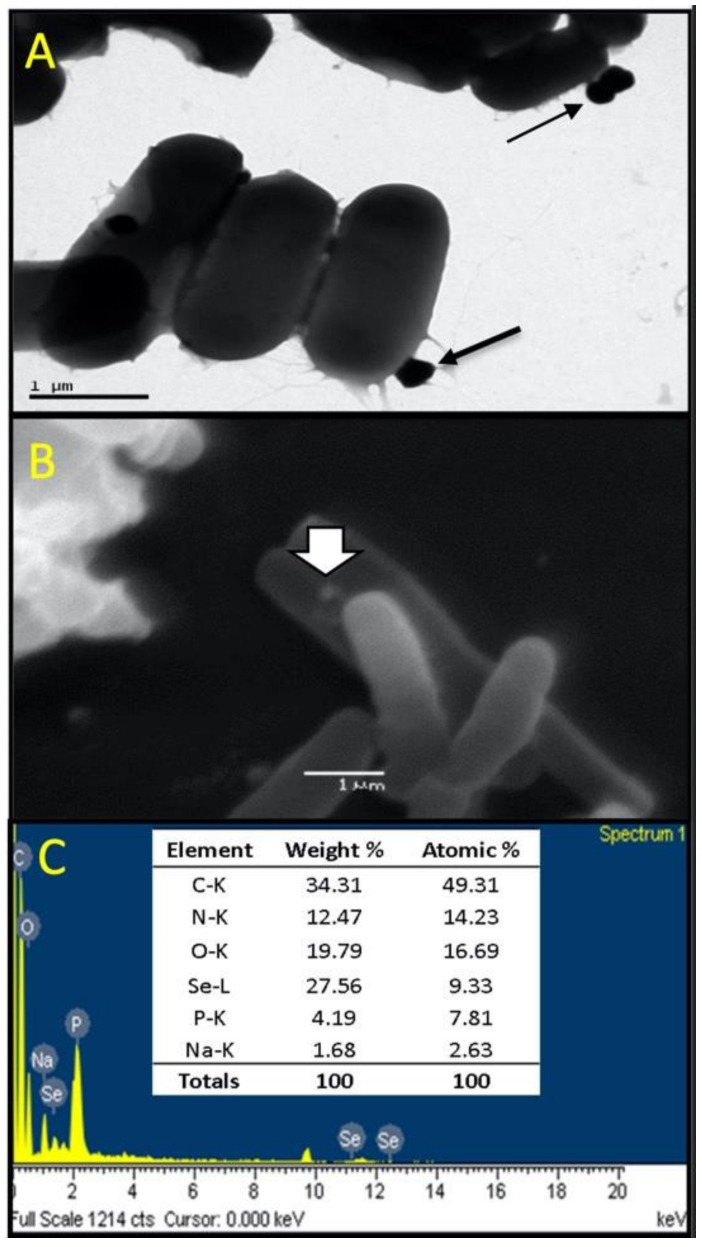
LABS14-Se^0^Nps. (**A**) TEM micrograph of LABS14 showing a bacillar morphology bacterium with nanoparticles attached to the cell surface (black arrows); (**B**) SEM micrograph showing a nanoparticle attached to the bacterial surface (white arrow); (**C**) SEM-EDS data confirmation of the presence of Se in the nanoparticles (Se^0^Nps).

TEM, SEM, and SEM-EDS observations (Figure 2) were performed to confirm that LABS14 was able to produce Se^0^Nps when Se was available in the culture medium. Electron microscopy revealed sphere-like nanoparticles, with sizes between 98 and 245 nm in diameter, attached to the surface of LABS14 cells (Figure 2A,B). The detection of Se in the nanoparticles was verified by SEM-EDS (Figure 2C), allowing them to be considered as Se^0^Nps. The presence of C, N, and O can be attributed to cell debris and the presence of Na and P could be associated to remnants of the culture medium (Figure 2C).

### 3.5. Molecular Identification of LABS14 Strain by 16S rDNA Sequence Analysis

LABS14 was identified by PCR, after amplifying its *16S rDNA* gene and sequencing the product of PCR (approximately 1500 bp) and subjecting them to a BLAST analysis. LABS14 strain was identified with a high level of confidence (98%) as *Lactiplantibacillus plantarum* (access number GenBank AY096004).

### 3.6. Effect of the Dietary Administration of Enriched-(LABS14-Se^0^Nps) as a Nutritional Supplement in Rainbow Trout (In Vivo Model)

#### 3.6.1. ROS in White Blood Cells and Lysozyme Activity in Plasma

The respiratory burst of the rainbow trout peripheral leukocytes was assessed by the reduction of NBT into formazan. Higher absorbances correspond to higher ROS concentrations.

**Figure 3:**
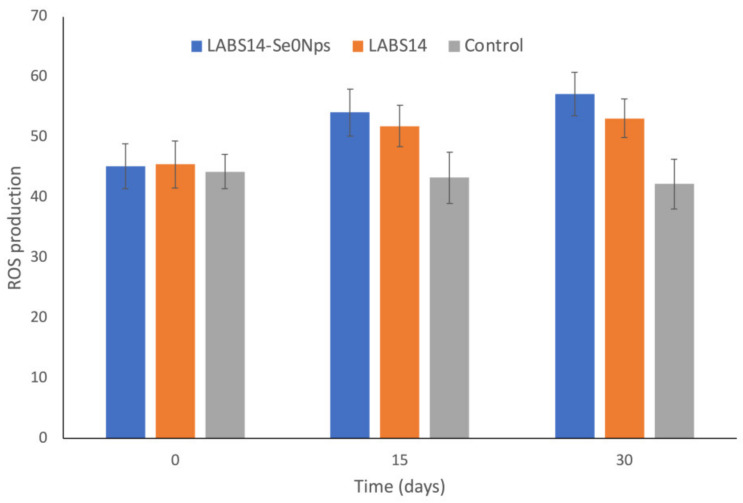
Reactive oxygen species (ROS) production by rainbow trout blood leukocytes, evaluated by nitroblue tetrazolium reduction into formazan. Fish food was supplemented for 30 days with 10^8^ CFU of Se nanoparticle-enriched *Lactiplantibacillus plantarum* S14 strain (LABS14-Se^0^Nps) g^−1^ or 10^8^ CFU *L. plantarum* S14 strain (LABS14) g^−1^. Control did not receive the bacterial strain. Bars indicate standard deviation.

On days 15 and 30, cellular ROS increased significantly in trout receiving LABS14-Se^0^Nps (diet D1) or LABS14 (diet D2) (*p* < 0.05) when compared to the control (Figure 3). On days 15 and 30, ROS concentration in the group LABS14-Se^0^Nps was higher, although not significantly, when compared to fish receiving LABS14 (*p* > 0.05) (Figure 3).

**Figure 4:**
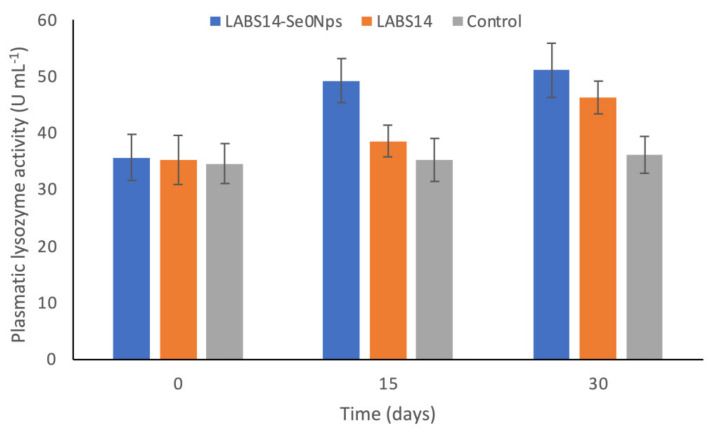
Plasmatic lysozyme activity (U mL^−1^) in rainbow trout whose food was supplemented, for 30 days, with 108 CFU of Se nanoparticle-enriched *Lactiplantibacillus plantarum* S14 strain (LABS14-Se^0^Nps) g^−1^ or 108 CFU *L. plantarum* S14 strain (LABS14) g^−1^. Control did not receive the bacterial strain. Bars indicate standard deviation.

Plasmatic lysozyme activity increased significantly (*p* < 0.05) in rainbow trout fed LABS14-Se^0^Nps supplemented food (diet D1) on days 15 and 30 when compared to the control group, while LABS14 (diet D2) achieved this only on day 30 (Figure 4). LABS14-Se^0^Nps supplementation caused the highest levels of plasmatic lysozyme activity on days 15 and 30, but only on day 15 was it significantly elevated (*p* < 0.05) when compared to that caused by LABS14 (Figure 4).

#### 3.6.2. Activity of the Antioxidant Enzyme Gpx

**Figure 5:**
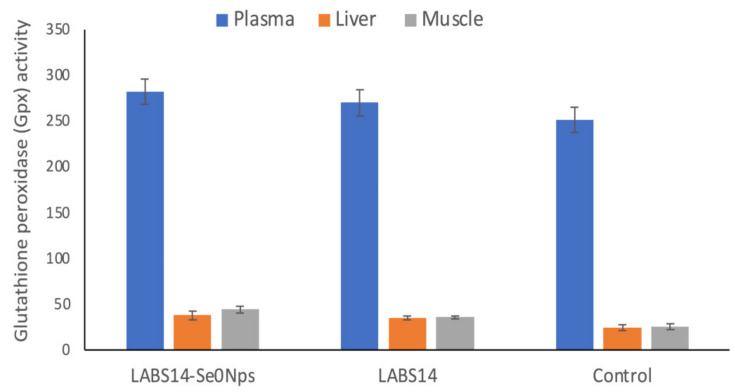
Glutathione peroxidase (mU mg^−1^ protein) activity in rainbow trout whose food was supplemented, for 30 days, with 10^8^ CFU of Se nanoparticle-enriched *Lactiplantibacillus plantarum* S14 strain (LABS14-Se^0^Nps) g^−1^ or 10^8^ CFU *L. plantarum* S14 strain (LABS14) g^−1^. Control did not receive the bacterial strain. Bars indicate standard deviation.

The Gpx activity in the plasma, liver, and dorsal muscle of rainbow trout administered LABS14-Se^0^Nps or LABS14 supplemented food for 30 days is shown in Figure 5. Significant increases in Gpx activity were observed on day 30 in the plasma, liver, and dorsal muscle in the groups receiving diets D1 or D2 (*p* < 0.05) when compared to the control. When compared to fish which received the diet D2, the group receiving the D1 diet showed higher Gpx activities in all samples, but the difference was significant only in plasma and muscle.

#### 3.6.3. Growth Performance and Survival

**Figure 6:**
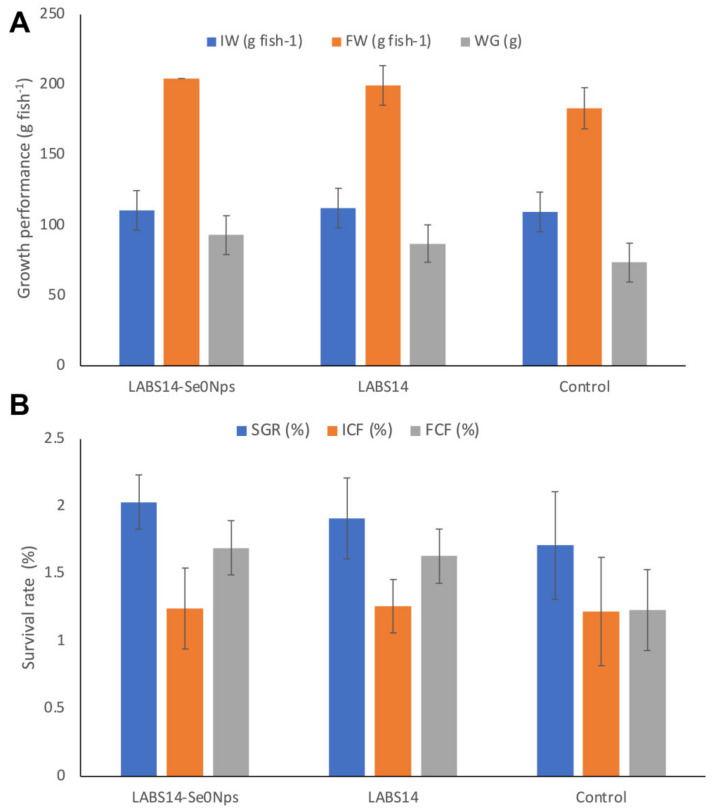
Growth performance. (**A**) initial weight (IW), final weight (FW) and weight gain (WG). (**B**) specific growth rate (SGR), initial condition factor (ICF) and final condition factor (FCF) of rainbow trout whose food was supplemented, for 30 days, with 10^8^ CFU of Se nanoparticle-enriched *Lactiplantibacillus plantarum* S14 strain (LABS14-Se^0^Nps) g^−1^ or 10^8^ CFU *L. plantarum* S14 strain (LABS14) g^−1^. Control did not receive the bacterial strain. Bars indicate standard deviation.

The growth and survival of fish receiving diet D1 or D2 for 30 days are shown in Figure 6A,B. Weight gain (WG) was significantly higher in the D1 (93.2 g) and D2 (87.1 g) fed groups when compared to the AD group (73.8 g) (*p* < 0.05). Similarly, the specific growth rate (SGR) values were significantly higher in D1 (2.03%) and D2 (1.91%) dietary treatment groups when compared to the control (1.71%) (*p* < 0.05). Although fish with D1 diet showed the highest SGR value, this was non-significantly higher than that of the group fed the diet D2.

The final condition factor (FCF) of rainbow trout administered the diet D1 (1.69%) was significantly higher than the FCF of the control group (1.23%) (*p* < 0.05) but non significantly higher than the group fed with the D2 diet (1.63%) (*p* > 0.05).

## 4. Discussion

Potential probiotic bacteria for aquaculture species, as reported by Merrifield et al. [18], should be naturally occurring and non-pathogenic in the natural habitat of the host, be easy to culture, and able to grow in the intestine of the host (thus, resist bile salts and low pH, adhere within the intestinal mucus, and capable to colonize the epithelial surface of the intestine). Probiotic candidates should lack plasmid-encoded genes providing antibiotic resistance and should have positive effects on the health and/or nutrition of fish. Dietary supplementation with probiotics contributes to the balance of the intestinal microbiota and probiotics have been proposed as an alternative to chemotherapeutants and antibiotics to avoid disease outbreaks, to mitigate the negative effects of stress, and to strengthen the antioxidant capacity and the immune system of fish [84]. Since intensive fish farming faces heavy losses caused by disease, probiotics are being used to control diseases [85].

LAB, such as *L. plantarum*, are administered as probiotics in fish production because of their positive effects, e.g., in feed utilization [86,87], as growth promoters [88], as immune response enhancers [89], and as stress tolerance improvers [86]. It has also been reported that some LAB are able to absorb Se ions and produce Se^0^Nps to become selenium-enriched bacterial cells with high biological activity [90,91,92]. On this basis, we hypothesized that supplementing fish food with a LAB strain having properties of a probiotic and, concurrently, able to biosynthesize Se^0^Nps may have a better potential to improve the innate immune response, the oxidative status, and productive parameters of rainbow trout than supplementing their food with only probiotics.

We obtained 16 possible LAB autochthonous isolates from the intestinal content of rainbow trout and tested them to select the most suitable probiotic LAB candidates. Only six of them (LABS2, LABS4, LABS8, LABS12, LABS13, and LABS14) showed characteristics attributable to LAB strains (rod-shaped, non-motile, Gram-positive, catalase negative and lactic acid producing bacteria) [93]. Therefore, these six LAB strains were considered to be tested, according to Rondón et al. [55], to determine if they possessed the characteristics of potential probiotics. The characteristics evaluated were their antibacterial activity, antibiotic susceptibility, viability at a low pH, tolerance to bile salts, hemolytic activity, and hydrophobicity. Strains not fulfilling the requirement of a particular characteristic were excluded and not considered to evaluate the remaining characteristics in them.

Results of the antibacterial activity showed that, except LABS12, all other strains (LABS2, LABS4, LABS8, LABS13, and LABS14) had antibacterial activity against Gram-positive (*S. aureus* and *B. subtilis*) and Gram-negative (*E. coli* and *P. aeruginosa*) indicator bacteria. Gram-positive indicator strains were more susceptible than Gram-negative indicator strains to the antibacterial activity of the isolated LAB, results akin to those described by Savadogo et al. [94] and Tebyanian et al. [95], indicating that Gram-negative bacteria are less susceptible than Gram-positive ones to the antibacterial mechanisms executed by LAB strains. The antibacterial activity of probiotics is presently mostly attributed to the production of antibacterial substances or metabolites and to competitive exclusion, i.e., competition with pathogens for nutrients and attachment sites, preventing pathogens from colonizing the intestine [96]. Antibacterial substances are produced by probiotic strains, organic acids (particularly lactic and acetic acids), hydrogen peroxide, and bacteriocins [97].

The European Food Safety Authority (EFSA) guidelines indicate that all bacterial strains intended for human or animal consumption must be tested for antibiotic susceptibility. The rationality is to avoid these bacteria to become a source of antibiotic resistance genes that may be transferred to other bacteria of the host microbiota or to environment bacterial communities [98]. According to Bujnakova and Strakova [99], strains harboring acquired resistance patterns must be excluded from hosts and environment. Our results concerning the susceptibility of LABS2, LABS4, LABS8, LABS13, and LABS14 to GEN, TET, OXY, ERY, FLO, and AMP, according to the CLSI [59], revealed that all five LAB strains, being susceptible to the antibiotics tested, were considered for the following tests, including hemolytic activity, hydrophobicity (which estimates the capacity of a cell to adhere to another cell surface), cell viability at low pH, and tolerance to bile salts. These tests evaluated, according to Rondón et al. [55], properties of the LAB isolates consistent with a probiotic.

The hemolytic activity assays showed that only LABS4 and LABS14 strains caused gamma hemolysis. In general, pathogenic bacteria are able to lyse erythrocytes and other cell membranes synthetizing and secreting hemolysins, considered as virulence factors [100]. Therefore, both alpha- and beta-hemolytic phenotypes are considered virulence-associated determinants of bacterial species and/or strains [101]. On the other hand, gamma-hemolysis indicates the lack of hemolytic activity of bacteria [102]. Thus, when considering the safety of a probiotic, the absence of hemolysins is an important consideration [14]. Hence, only LABS4 and LABS14 strains could be reasonably selected for the following test: hydrophobicity assay.

Cell hydrophobicity, important property for probiotic bacteria, allows their adherence, including that to the epithelium of the intestine and to colonize the gastrointestinal tract to provide their beneficial effects, such as exclusion of enteropathogenic bacteria [103,104,105]. LABS14 strain showed a higher level of hydrophobicity than LABS4; nevertheless, the hydrophobicity of both strains was considered as medium. Hydrophobicity figures from 30% to 60% are considered as medium while over 60% is considered as high [62], indicating that besides hydrophobicity other variables may influence the adherence of cells. Therefore, it is reasonable to consider that LABS4 and LABS14 strains could efficiently avoid the adherence of enteropathogenic bacteria to the gastrointestinal epithelium of rainbow trout. Thus, LABS4 and LABS14 strains were both included in the following test: cell viability of the LAB strains isolated at a low pH assay.

According to Bravo et al. [106], in salmonid fish fed with artificial diets, the gastric pH drops to 3.5. At a pH < 5, the growth of several Gram-negative bacteria is reduced, so a low pH also creates a natural barrier against pathogens from the environment and favors the proliferation of acid-tolerant, beneficial bacteria, such as LAB [107]. LABS14 and LABS4 strains were able to tolerate a pH of 3 (74.8% and 57.1% viability, respectively). Based on the report of Fečkaninová et al. [93], LABS14 and LABS4 viabilities after being grown at a low pH suggest that these strains can reach the intestine in a viable form. Therefore, LABS14 and LABS4 were both included in the following test: tolerance of the LAB strains isolated to bile salts assay.

Bacterial resistance to intestinal bile salts is another parameter to consider when selecting probiotic bacteria [108]. Then, we can suggest that LABS14 and LABS4 may colonize the rainbow trout intestine because both resisted 0.3% bile salts, a concentration similar to that resisted by a strain of *L. plantarum* with probiotic potentiality obtained from the Mediterranean trout (*Salmo macrostigma*) intestine [109]. The secretion of bile salts into the gastrointestinal tract can hinder the growth of bacteria, acting as antibacterial molecules, even if they are present at low concentrations [110]. Nevertheless, probiotics may tolerate them producing bile salts hydrolase enzymes [111]. Thus, the capacity of LABS14 and LABS4 to produce Se^0^Nps when cultured in Na_2_SeO_3_ supplemented culture medium was evaluated.

The metabolism of LAB leading to transform inorganic Se into elemental Se (Se^0^) involves a high activity of the enzyme glutathione reductase (GR) [112] and these authors concluded that a number of LAB strains are overexpressed genes coding for GR (*GshR*/*gor*) when grown in the presence of Na_2_SeO_3_. Selenite may interact with glutathione to produce selenotrisulfide derivatives, which participate in the conversion of inorganic Se into bioactive selenocompounds. These conversions allow reduced glutathione (GSH) to be oxidized to Se diglutathione (GSSeSG), which is then reduced again to GSH by GR. GSSeSG is decomposed to produce Se^0^ [113], allowing LAB cells to form Se^0^-cysteine (SeCys) and Se^0^-metionine [41].

When cultured in the presence of Na_2_SeO_3_, LABS14 colonies, but not LABS4 colonies, acquired a red color. Daza et al. [66] and Ravanal [114] also reported the same phenomenon for the Se^0^Nps biosynthesized by the bacterium *Pantoea agglomerans*, attributable to intracellular red amorphous Se, a non-crystalline allotropic form of Se, resulting from the enzymatic reduction of Na_2_SeO_3_. The production of Se^0^Nps by bacteria would be a detoxification process permitting Se (IV) to be reduced to insoluble Se (Se^0^) and later stored as electron-dense amorphous granules which can be detected in the cytoplasm and/or extracellularly [115,116]. Since LABS4 was unable to reduce Na_2_SeO_3_, only LABS14 strain cells were analyzed by TEM, SEM, and SEM-EDS. Observations revealed that LABS14 was able to produce sphere-like Se^0^Nps, which were located in the surface of cells.

The transformation of inorganic Se into Se^0^Nps by LAB has been previously reported [40,41,117,118]. Reported size of biosynthesized Se^0^Nps by some *L. plantarum* strains are: <250 nm [116], between 55 and 90 nm [115] and 142.6 nm average [42]. According to Zhang et al. [117], smaller Se^0^Nps have greater biological activity. Hosnedlova et al. [118] showed that the absorption of Se^0^Nps synthesized by *L. lactis* in the gastrointestinal tract was 15–250 times higher when particles were 50–90 nm compared to nanoparticles of 125–155 nm.

Since LABS14 showed that its tested characteristics are consistent with those of probiotics and that it was concurrently able to produce Se^0^Nps, it was molecularly identified by 16S rDNA sequence analysis. The analysis revealed, with 98% of confidence, that this isolate (GenBank accession number AY096004.1) corresponds to *L. plantarum*, a LAB species of considerable industrial and medical interest [119] and already evaluated as a probiotic for rainbow trout [27,120,121].

In the present study, *L. plantarum* strain S14 containing Se^0^Nps (LABS14-Se^0^Nps) or lacking Se^0^Nps (LABS14), corresponding to diet D1 or D2, respectively, were administered to rainbow trout as a food supplement for 30 days. The effects of supplementations on two parameters of the innate immune response (ROS production by WBC and plasmatic lysozyme), oxidative status (activity of the enzyme Gpx in plasma, liver and muscle), and in the productive parameters were studied.

Phagocytosis allows to ingest and eliminate microbial pathogens and apoptotic cells by phagocytes [122]. Phagocytes produce respiratory bursts to eliminate pathogens and these bursts can be measured to evaluate the defensive response of the host. Superoxide anions, together with hydroxyl radicals and nitric oxides, which are inducible ROS, enhance the microbicidal capacity of phagocytes [123,124]. Our results showed a significantly higher ROS concentration in WBCs of rainbow trout fed with D1 or D2 diets since day 15. The last day of the assay (day 30) ROS concentrations were even higher than those of day 15, showing a progressive improvement of the microbicidal capacity of WBCs. Soltani et al. [125] demonstrated that administering *L. plantarum* in the diet also improved neutrophil ROS concentration in carps *(Cyprinus carpio*).

Although a non-significant difference, WBCs of fish fed with D1 diet showed a higher ROS concentration than those of fish feed with the D2 diet on days 15 and 30. Hence, a combined effect of probiotic and Se^0^Nps on the respiratory burst of rainbow trout phagocytic cells can be suggested. Chen et al. [126], showed a potential higher phagocytic capacity of macrophages of mice fed with *Lactobacillus* sp. Containing higher concentrations of intracytoplasmic Se^0^, with their phagocytic index depending on the intracellular Se concentration in Se-enriched bacteria. They pointed out that Se might provide additional protection to the cell membrane of macrophages against oxidative damage.

Lysozyme, produced by phagocytic cells and secreted into blood and mucus to produce bacteriolytic activities, is a very significant nonspecific immune factor in fish [127]. Its level and activity in fish depends, among others, on the nutritional status [128]. This study demonstrated that rainbow trout receiving D1 or D2 diets significantly improved plasmatic lysozyme activity levels on day 15 when compared to the control group. Noteworthily, D1 diet caused, although not significantly, a better lysozyme activity than D2 treatment on both testing days, suggesting a combined effect of the probiotic strain LABS14 and Se^0^Nps on lysozyme plasmatic level by rainbow trout.

The effect of food supplemented with *L. plantarum* on the increasing of lysozyme activity in the blood has been previously demonstrated in the rainbow trout [129], striped catfish (*Pangasianodon hypophthalmus*) [130], and the Orange-spotted grouper (*Epinephelus coioides*) [131]. Furthermore, other reports demonstrated that the dietary supplementation with Se^0^Nps significantly improved tissular lysozyme activity in various fish species, including, among others, rainbow trout [81,132], yellowtail kingfish (*Seriola lalandi*) [133], and Nile tilapia (*Oreochromis niloticus*) [134]. We have been unable to find studies pertaining the combined effect of *L. plantarum* and Se^0^Nps on lysozyme activity in vertebrates. However, Shang et al. [135] reported a significantly higher activity of blood lysozyme in carps (*Cyprinus carpio* var. *specularis*) exposed to Hg for 30 days when their food was supplemented for 30 days with Se-enriched, not in the form of nanoparticles, probiotic *Bacillus subtilis*.

Antioxidants are important for fish in wildlife, but also in captivity, due to various stressors, such as variations of the oxygen levels associated to an increase of ROS [136]. Magnoni et al. [137] indicated that both nutritional and environmental stressors are able to cause OS, reduce innate immune and OS response, and diminish energy generation by affecting metabolic pathways in rainbow trout. In our study, at the end of the 30-day assay, diets D1 and D2 caused higher Gpx activity in plasma, liver, and muscle of the rainbow trout when compared the control. On the other hand, diet D1 also improved all three tested Gpx activities when compared to D2, but significantly only for plasmatic and muscle, suggesting a combined effect of LABS14 strain and the Se^0^Nps produced by the bacterium on tissular Gpx activity. Chen et al. [126] reported that supplementation of mice food with Se-enriched *Lactobacillus* ameliorated or enhanced Gpx activity biosynthesis in a *Lactobacillus* cytoplasm Se concentration-dependent manner. Moreover, Mengistu et al. [138] reported a combined effect of Se and *Lactobacillus acidophilus*. Supplementation of chickens’ food with Se-enriched *Lactobacillus acidophilus* for 42 days determined a significant upregulation of the Gpx1, Gpx4, seleno-protein W, and interferon gamma mRNA expression when compared to mRNA expression in groups of chickens feed with *L. acidophilus* or sodium selenite supplemented food and control group (non-supplemented food). Shang et al. [43] also demonstrated an increase of Gxp activity in juvenile *Luciobarbus capito* using Se-enriched *L. plantarum* as a food supplement. Shang and colleagues did not precisely clarify what form of Se was present in the cytoplasm of *L. plantarum*.

This study also investigated the effect of D1 or D2 diets as a 30-day food supplement on growth performance. Both diets significantly improved all growth performance indexes evaluated when compared to the control. Nevertheless, the comparison of nutrient supplementations showed that rainbow trout fed with diet D1 showed a higher, but non-significant, growth performance than the one achieved by diet D2. A FCF above 1.00 indicates a good health condition (well-being) and it is associated to the improvement of important production parameters, including fertility rate, and the production of high-quality gametes [139]. The results of the present work suggest a combined effect of LABS14 strain and Se^0^Nps on physiological processes that impact on the rainbow trout growth performance. Diet D1 may allow rainbow trout to be more efficient to accumulate reserves of energy.

Probiotic bacteria shield epithelial cells from toxins produced by pathogenic microorganisms exerting direct antibacterial action toward competing enteropathogens, which may prevent colonization by pathogens [140], favorins intestinal absorption, and immunity [141]. The nutrients absorbed (vitamins, amino acids, and fatty acids) reach the bloodstream with ease, contributing to the metabolic functions of the whole body. Thus, host derived probiotics can enhance nutrients digestibleness in the intestine of fish, competing with pathogenic microorganisms and inhibiting their detrimental impact on the intestinal wellbeing [142]. The use of diets supplemented with *L. plantarum* has been shown to improve food conversion and growth in a number of fish species [133,143,144,145,146]. Soltani et al. [125] indicated the improvement of immunological parameters achieved when supplying *L. plantarum* in the diet, resulting in better growth conditions for rainbow trout. The beneficial effect of Se^0^Nps on the growth performance of fish, such as, among others, the European seabass (*Dicentrarchus labrax*) [147], the Nile tilapia (*Oreochromis niloticus*) [31], and the crucian carp (*Carassius auratus gibelio*) [148], was reported. A previous study using biogenic Se^0^Nps as a dietary supplement in rainbow trout showed an increase of the final condition factor index after only 30 days of experimentation [132]. A *Bacillus subtilis* selenium-enriched probiotic, administered in the diet as a growth promoter, has been evaluated in broiler chicken with promising results [149].

## 5. Conclusions

Our results reported the isolation of a LAB strain, named S14 strain, from a sample of intestinal content obtained from healthy rainbow trout. S14 strain was identified, with a high level of confidence (98%), as a member of the *Lactiplantibacillus plantarum* species. This strain showed characteristics typically present in probiotics and, concurrently, the capacity to biosynthesize Se^0^Nps. The supplementation of fish diet with LABS14-Se^0^Nps for 30 days significantly improved respiratory burst and plasmatic lysozyme (innate immune response), as well as glutathione peroxidase (GPX) (oxidative status) activities and productive parameters when compared to controls. Moreover, it improved those parameters when compared to fish receiving LABS14, but significant only for plasmatic and muscle GPX. Therefore, we propose LABS14-Se^0^Nps as a promising alternative for nutritional supplementation for rainbow trout or even other salmonids.

## Figures and Tables

**Table 1:** Antibacterial susceptibility standards for testing, by the disc diffusion antibiotic method [59] and, classification of antibiotics [60] used in this study.

Antibiotic (µg)	Interpretive Categories and Inhibition Diameter Breakpoints, Nearest Whole mm			Antibiotic Class	Mechanism of Action
	S	I	R		
GEN (10)	≥15	14–13	≤12	Aminoglycosides	Inhibitor of protein synthesis
TET (30)	≥15	14–12	≤11	Tetracycline	Inhibitor of protein synthesis
OXY (30)	≥15	14–12	≤11	Tetracycline	Inhibitor of protein synthesis
ERY (15)	≥22	21–16	≤15	Macrolides	Inhibitor of protein synthesis
FLO (30)	≥19	18–15	≤14	Amphenicols	Inhibitor of protein synthesis
AMP (10)	≥17	16–14	≤13	Β-Lactams	Inhibitor of the cell wall synthesis

GEN: gentamicin; TET: tetracycline; OXY: oxytetracycline; ERY: erythromycin; FLO: florfenicol; AMP: ampicillin. S: susceptible; I: intermediate; R: resistant.

**Table 2:** Composition of the commercial extruded food used as acclimation diet (AD).

Compound	%	Compound	%	Compound	%
Crude protein	39–43%	Lipids	10–16%	Ash	9–12%
Moisture	7–13%	Calcium	1–2%	Fiber	3–4%
Phosphate	1–1.4%				

**Table 3:** Morphological characteristics and catalase activity of 16 isolates obtained from the intestinal content of rainbow trout.

Isolate	Gram	Morphology	Catalase
S1	positive	coccoid	positive
S2	positive	rod-shaped	negative
S3	positive	coccoid	positive
S4	positive	rod-shaped	negative
S5	positive	coccoid	positive
S6	positive	coccoid	positive
S7	negative	rod-shaped	N/A
S8	positive	rod-shaped	negative
S9	negative	rod-shaped	N/A
S10	positive	coccoid	positive
S11	positive	coccoid	positive
S12	positive	rod-shaped	negative
S13	positive	rod-shaped	negative
S14	positive	rod-shaped	negative
S15	positive	rod-shaped	positive
S16	positive	rod-shaped	negative

Isolates were cultured in MRS agar 24–48 h at 37 °C under microaerobic conditions. N/A: Not Applicable.

**Table 4:** Fermentation pattern of hexoses (glucose, fructose or galactose) or pentoses (ribose, xylose or arabinose) of isolates S2, S4, S8, S12, S13 and S14 obtained from the intestinal content of rainbow trout when cultured in MRS broth plus phenol red.

Isolate	Carbohydrate					
	Glucose	Fructose	Galactose	Ribose	Xylose	Arabinose
S2	+	+	−	−	−	−
S4	+g	+g	−	−	−	−
S8	+g	+g	−	+	+	+
S12	+g	+g	−	+	+	+
S13	+	+	−	−	−	−
S14	+	+g	−	+	+	+

Detection of carbohydrate fermentation: + (positive): fermentation of the carbohydrate, − (negative): no fermentation of the carbohydrate, g: presence of gas in Durham tube.

**Table 5:** Antibacterial activity of LAB strains (LABS2, LABS4, LABS8, LABS12, LABS13 and LABS14) isolated from the intestinal content of rainbow trout against indicator strains *Staphylococcus aureus* ATCC 25923, *B. subtilis* ATCC 6633, *E. coli* ATCC 25922 and *P. aeruginosa* ATCC 10145.

LAB Strains	Inhibition Halo (in mm) of Reference Bacterial Strains			
	*S. aureus* ATCC 25923	*B. subtilis* ATCC 6633	*E. coli* ATCC 25922	*P. aeruginosa* ATCC 10145
LABS2	16.7 ± 0.9 ^a^	18.9 ± 0.4 ^a^	18.6 ± 1.0 ^a^	13.9± 0.5 ^a^
LABS4	30.8 ± 0.7 ^b^	25.3 ± 0.7 ^b^	16.2 ± 0.3 ^b^	31.6± 0.8 ^b^
LABS8	20.8 ± 0.4 ^c^	18.3 ± 0.4 ^a^	8.9 ± 0.4 ^c^	12.6 ± 0.4 ^a^
LABS12	-	12.8 ± 0.7 ^c^	-	-
LABS13	20.5 ± 0.8 ^c^	16.5 ± 0.7 ^d^	11.5 ± 0.4 ^d^	12.4 ± 1.1 ^a^
LABS14	26.3 ± 0.4 ^d^	20.9 ± 1.1 ^e^	12.9 ± 0.3 ^de^	18.9 ± 0.3 ^c^

-: inhibition halo not observed. a–e Means with different superscripts within a column are significantly different (*p* < 0.05). Values are means of two independent experiments, each in triplicate.

**Table 6:** Type of hemolytic activity on MRS agar plus 5% human blood after 24 h, 48 h and 72 h incubation at 37 °C of the selected LAB strains obtained from the intestinal content of rainbow trout.

LAB Strain	Type of Hemolysis		
	24 h	48 h	72 h
LABS2	Alpha	Alpha	Alpha
LABS4	Gamma	Gamma	Gamma
LABS8	Alpha	Alpha	Alpha
LABS13	Beta	Beta	Beta
LABS14	Gamma	Gamma	Gamma

The experiments were carried out in triplicate.

**Table 7:** Tolerance to acidic pH and 0.3% bile salts evaluated by the viability of LABS4 and LABS14 strains isolated from rainbow trout intestinal content, incubated for 4 h at 37 °C at pH 3.0 or in the presence of 0.3% (*w*/*v*) bile salts.

LAB Strain	Acid Resistance		Bile Salts Resistance	
	pH 3 (Viability %)	Control	Bile Salt (Viability %)	Control
LABS4	* 8 × 10^8^ ± 0.06 (57.1)	14 × 10^8^ ± 0.12	* 9 × 10^8^ ± 0.07 (69.2)	13 × 10^8^ ± 0.18
LABS14	* 12 × 10^8^ ± 0.10 (74.8)	16 × 10^8^ ± 0.09	* 12 × 10^8^ ± 0.14 (82.3)	15 × 10^8^ ± 0.03

Acid resistance: N° of CFU mL^−1^; Bile resistance: N° of CFU mL^−1^. Results are shown as viable cells percentage when compared to cells not subjected to HCl or bile salts (control) assigned as 100%. * Significantly smaller than corresponding control (*p* < 0.05). Data is given as mean ± SD; Experiments were carried out in triplicate.

## Data Availability

Not applicable.

## References

[1] Iversen A., Asche F., Hermansen Ø., Nilstøyl R. (2020). Production cost and competitiveness in major salmon farming countries 2003–2018. Aquaculture.

[2] Valenzuela C.A., Ponce C., Zuloaga R., González P., Avendaño-Herrera R., Valdés J.A., Molina A. (2020). Effects of crowding on the three main proteolytic mechanisms of skeletal muscle in rainbow trout (*Oncorhynchus mykiss*). BMC Vet. Res..

[3] Harper C., Wolf J.C. (2009). Morphologic Effects of the Stress Response in Fish. ILAR J..

[4] Subpesca (2017). Informe Ambiental de la Acuicultura.

[5] Urbina M.A. (2016). Temporal variation on environmental variables and pollution indicators in marine sediments under sea Salmon farming cages in protected and exposed zones in the Chilean inland Southern Sea. Sci. Total Environ..

[6] Cabello F.C., Godfrey H.P. (2019). Salmon aquaculture, *Piscirickettsia salmonis* virulence, and One Health: Dealing with harmful synergies between heavy antimicrobial use and piscine and human health. Aquaculture.

[7] Talwar C., Nagar S., Lal R., Negi R.K. (2018). Fish gut microbiome: Current approaches and future perspectives. Indian J. Microbiol..

[8] Navarrete P., Mardones P., Opazo R., Espejo R., Romero J. (2008). Oxytetracycline Treatment Reduces Bacterial Diversity of Intestinal Microbiota of Atlantic Salmon. J. Aquat. Anim. Health.

[9] Donati V.L., Madsen L., Middelboe M., Strube M.L., Dalsgaard I. (2022). The Gut Microbiota of Healthy and *Flavobacterium psychrophilum*-Infected Rainbow Trout Fry Is Shaped by Antibiotics and Phage Therapies. Front. Microbiol..

[10] Valdés N., González A., García V., Tello M. (2020). Analysis of the Microbiome of Rainbow Trout (*Oncorhynchus mykiss*) Exposed to the Pathogen *Flavobacterium psychrophilum* 10094. Microbiol. Resour. Announc..

[11] Wang N., Jiang M., Zhang P., Shu H., Li Y., Guo Z., Li Y. (2020). Amelioration of Cd-induced bioaccumulation, oxidative stress and intestinal microbiota by *Bacillus cereus* in *Carassius auratus gibelio*. Chemosphere.

[12] Wang N., Yuan Z., Wang K., Gao D., Liu Z., Liles M.R. (2019). Consumption of florfenicol-medicated feed alters the composition of the channel catfish intestinal microbiota including enriching the relative abundance of opportunistic pathogens. Aquaculture.

[13] Sernapesca Informe Sobre Uso de Antimicrobianos en la Salmonicultura Nacional 2019. Valparaíso: Servicio Nacional de Pesca y Acuicultura 2020. http://www.sernapesca.cl.

[14] FAO/WHO (2006). Probiotics in Food. Health and Nutritional Properties and Guidelines for Evaluation.

[15] Sharifuzzaman S.M., Austin B., Austin B., Newaj-Fyzul A. (2017). Probiotics for disease control in aquaculture. Diagnosis and Control of Diseases of Fish and Shellfish.

[16] Das S., Mondal K., Haque S. (2017). A review on application of probiotic, prebiotic and synbiotic for sustainable development of aquaculture. J. Entomol. Zool. Stud..

[17] Carvalho E.D., David G.S., Silva R.J. (2012). Health and Environment in Aquaculture.

[18] Merrifield D., Bradley G., Harper G., Baker R., Munn C., Davies S. (2011). Assessment of the effects of vegetative and lyophilized *Pediococcus acidilactici* on growth, feed utilization, intestinal colonization and health parameters of rainbow trout (*Oncorhynchus mykiss* Walbaum). Aquac. Nutr..

[19] Lamari F., Castex M., Larcher T., Ledevin M., Mazurais D., Bakhrouf A., Gatesoupe F.-J. (2013). Comparison of the effects of the dietary addition of two lactic acid bacteria on the development and conformation of sea bass larvae, *Dicentrarchus labrax*, and the influence on associated microbiota. Aquaculture.

[20] Shahid M., Hussain B., Riaz D., Khurshid M., Ismail M., Tariq M. (2016). Identification and partial characterization of potential probiotic lactic acid bacteria in freshwater *Labeo rohita* and *Cirrhinus mrigala*. Aquac. Res..

[21] Quinto E.J., Jiménez P., Caro I., Tejero J., Mateo J., Girbés T. (2014). Probiotic Lactic Acid Bacteria: A Review. Food Nutr. Sci..

[22] Olofsson T.C., Butler È., Markowicz P., Lindholm C., Larsson L., Vásquez A. (2016). Lactic acid bacterial symbionts in honeybees—An unknown key to honey’s antimicrobial and therapeutic activities. Int. Wound J..

[23] Llewellyn M.S., McGinnity P., Dionne M., Letourneau J., Thonier F., Carvalho G.R., Creer S., Derome N. (2016). The biogeography of the Atlantic salmon (Salmo salar) gut microbiome. ISME J..

[24] Fidanza M., Panigrahi P., Kollmann T.R. (2021). *Lactiplantibacillus plantarum*–Nomad and Ideal Probiotic. Front. Microbiol..

[25] Gildberg A., Johansen A., Bøgwald J. (1995). Growth and survival of Atlantic salmon (*Salmo salar*) fry given diets supplemented with fish protein hydrolysate and lactic acid bacteria during a challenge trial with *Aeromonas salmonicida*. Aquaculture.

[26] Pérez-Sánchez T., Balcázar J., García Y., Halaihel N., Vendrell D., De Blas I., Merrifield D., Ruiz-Zarzuela I. (2011). Identification and characterization of lactic acid bacteria isolated from rainbow trout, *Oncorhynchus mykiss* (Walbaum), with inhibitory activity against *Lactococcus garvieae*. J. Fish Dis..

[27] Soltani M., Pakzad K., Taheri-Mirghaed A., Mirzargar S., Hosseini S.P., Yosefi P., Soleymani N. (2019). Dietary Application of the Probiotic *Lactobacillus plantarum* 426951 Enhances Immune Status and Growth of Rainbow Trout (*Oncorhynchus mykiss*) Vaccinated Against *Yersinia ruckeri*. Probiotics Antimicrob. Proteins.

[28] Takahashi K., Suzuki N., Ogra Y. (2020). Effect of gut microflora on nutritional availability of selenium. Food Chem..

[29] Naderi M., Keyvanshokooh S., Salati A.P., Ghaedi A. (2017). Combined or individual effects of dietary vitamin E and selenium nanoparticles on humoral immune status and serum parameters of rainbow trout (*Oncorhynchus mykiss*) under high stocking density. Aquaculture.

[30] Baeverfjord G., Prabhu P.A., Fjelldal P.G., Albrektsen S., Hatlen B., Denstadli V., Ytteborg E., Takle H., Lock E.-J., Berntssen M.H.G. (2018). Mineral nutrition and bone health in salmonids. Rev. Aquac..

[31] Rathore S.S., Murthy H.S., Mamun M.A.-A., Nasren S., Rakesh K., Kumar B.T.N., Abhiman P.B., Khandagale A.S. (2021). Nanoselenium supplementation to ameliorate nutrition physiology, immune response, antioxidant system and disease resistance against *Aeromonas hydrophila* in monosex Nile tilapia (*Oreochromis niloticus*). Biol. Trace Elem. Res..

[32] Ibrahim M.S., El-gendy G.M., Ahmed A.I., Elharoun E.R., Hassaan M.S. (2021). Nanoselenium versus bulk selenium as a dietary supplement: Effects on growth, feed efficiency, intestinal histology, haemato-biochemical and oxidative stress biomarkers in Nile tilapia (*Oreochromis niloticus* Linnaeus, 1758) fingerlings. Aquac. Res..

[33] Karamzadeh M., Yahyavi M., Salarzadeh A., Nokhbe Zare D. (2021). The effects of different concentrations of selenium and zinc nanoparticles on growth performance, survival and chemical composition of whiteleg shrimp (*Litopenaeus vannamei*). Iran. Sci. Fish. J..

[34] Deilamy Pour H., Mousavi S.M., Zakeri M., Keyvanshokooh S., Kochanian P. (2021). Synergistic effects of selenium and magnesium nanoparticles on growth, digestive enzymes, some serum biochemical parameters and immunity of Asian sea bass (*Lates calcarifer*). Biol. Trace Elem. Res..

[35] Sarkar B., Bhattacharjee S., Daware A., Tribedi P., Krishnani K.K., Minhas P.S. (2015). Selenium nanoparticles for stress-resilient fish and livestock. Nanoscale Res. Lett..

[36] Mechlaoui M., Dominguez D., Robaina L., Geraert P.-A., Kaushik S., Saleh R., Briens M., Montero D., Izquierdo M. (2019). Effects of different dietary selenium sources on growth performance, liver and muscle composition, antioxidant status, stress response and expression of related genes in gilthead seabream (*Sparus aurata*). Aquaculture.

[37] Arshad A. (2017). Bacterial Synthesis and Applications of Nanoparticles. Nano Sci. Nano Technol..

[38] Dawood M.A.O., Zommara M., Eweedah N.M., Helal A.I. (2020). The evaluation of growth performance, blood health, oxidative status and immune-related gene expression in Nile tilapia (*Oreochromis niloticus*) fed dietary nano selenium spheres produced by lactic acid bacteria. Aquaculture.

[39] Yang J., Yang H. (2021). Recent development in Se-enriched yeast, lactic acid bacteria and bifidobacterial. Crit. Rev. Food Sci. Nutr..

[40] Moreno-Martin G., Pescuma M., Pérez-Corona T., Mozzi F., Madrid Y. (2017). Determination of size and mass-and number-based concentration of biogenic SeNPs synthesized by lactic acid bacteria by using a multimethod approach. Anal. Chim. Acta.

[41] Pescuma M., Gomez-Gomez B., Perez-Corona T., Font G., Madrid Y., Mozzi F. (2017). Food prospects of selenium enriched-*Lactobacillus acidophilus* CRL 636 and *Lactobacillus reuteri* CRL 1101. J. Funct. Foods.

[42] Salama H.H., El-Sayed N., Abd-Rabou N.S., Hassan Z.M.R. (2021). Production and use of eco-friendly selenium nanoparticles in the fortification of yoghurt. J. Food Process. Preserv..

[43] Shang X., Xu W., Zhao Z., Luo L., Zhang Q., Li M., Sun Q., Geng L. (2022). Effects of exposure to cadmium (Cd) and selenium-enriched *Lactobacillus plantarum* in *Luciobarbus capito*: Bioaccumulation, antioxidant responses and intestinal microflora. Comp. Biochem. Physiol. Part C Toxicol. Pharmacol..

[44] Khattab A.E.-N., Darwish A.M., Othman S.I., Allam A.A., Alqhtani H.A. (2022). Anti-inflammatory and Immunomodulatory Potency of Selenium-Enriched Probiotic Mutants in Mice with Induced Ulcerative Colitis. Biol. Trace Elem. Res..

[45] Office International des Epizooties (OIE) (2015). Welfare of farmed fish during transport. OIE—Aquatic Animal Health Code.

[46] American Veterinary Medical Association (AVMA) (2020). Guidelines for the Euthanasia of Animals: 2020 Edition.

[47] Huys G., D’Haene K., Swings J. (2002). Influence of the culture medium on antibiotic susceptibility testing of food-associated lactic acid bacteria with the agar overlay disc diffusion method. Lett. Appl. Microbiol..

[48] Saha U.S., Misra R., Tiwari D., Prasad K.N. (2016). A cost-effective anaerobic culture method & its comparison with a standard method. Indian J. Med. Res..

[49] Temmerman R., Huys G., Swings J. (2004). Identification of lactic acid bacteria: Culture-dependent and culture-independent methods. Trends Food Sci. Technol..

[50] Khalid K. (2011). An overview of lactic acid bacteria. Int. J. Biosci..

[51] Procop G.W., Church D.L., Hall G.S., Janda W.M., Koneman E.W., Schreckenberger P.C., Woods G.L. (2017). Koneman’s Color Atlas and Textbook of Diagnostic Microbiology.

[52] Salvetti E., Torriani S., Felis G.E. (2012). The Genus Lactobacillus: A Taxonomic Update. Probiotics Antimicrob. Proteins.

[53] Klayraung S., Okonogi S. (2009). Antibacterial and Antioxidant activities of acid and bile resistant strains of *Lactobacillus fermentum* isolated from miang. Braz. J. Microbiol..

[54] Erkus O. (2007). Isolation, Phenotypic and Genotypic Characterization of Yoghurt Starter Bacteria. Master’s Thesis.

[55] Rondón A.J., Samaniego L.M., Bocourt R., Rodríguez S., Milián G., Ranilla M.J., Laurencio M., Pérez M. (2008). Aislamiento, identificación y caracterización parcial de las propiedades probióticas de cepas de *Lactobacillus* sp. procedentes del tracto gastrointestinal de pollos de ceba. Cienc. Tecnol. Aliment..

[56] Schillinger U., Lücke F. (1989). Antibacterial activity of *Lactobacillus* sake isolated from meat. Appl. Environ. Microbiol..

[57] Geria M., Dambrosio A., Normanno G., Lorusso V., Caridi A. (2014). Antagonistic activity of dairy lactobacilli against gram-foodborne pathogens. Acta Sci. Technol..

[58] Alonso S., Castro M.C., Berdasco M., García de la Banda I., Moreno-Ventas X., Hernández de Rojas A. (2019). Isolation and Partial Characterization of Lactic Acid Bacteria from the Gut Microbiota of Marine Fishes for Potential Application as Probiotics in Aquaculture. Probiotics Antimicrob. Proteins.

[59] (2022). Performance Standard for Antimicrobial Susceptibility Testing.

[60] Tripathi K.D., Tripathi M. (2019). Antimicrobial drugs, Section 12. Essentials of Medical Pharmacology.

[61] Rodrigues L., Fortes L.L., Durmaz E., Goh Y.J., Sanozky-Dawes R.B., Klaenhammer T.R. (2012). Characterization of *Lactobacillus gasseri* isolates from a breast-fed infant. Gut Microbes.

[62] Xu H., Jeong H.S., Lee H.Y., Ahn J. (2009). Assessment of cell surface properties and adhesion potential of selected probiotic strains. Lett. Appl. Microbiol..

[63] Sánchez-Ortiz A.C., Luna-González A., Campa-Córdova A.I., Escamilla-Montes R., Flores-Miranda M., Mazón-Suástegui J.M. (2015). Isolation and characterization of potential probiotic bacteria from pustulose ark (*Anadara tuberculosa*) suitable for shrimp farming. Lat. Am. J. Aquat. Res..

[64] Kaushik J., Kumar A., Duary R.K., Mohanty A.K., Grover S., Batish V.K. (2009). Functional and Probiotic Attributes of an Indigenous Isolate of *Lactobacillus plantarum*. PLoS ONE.

[65] Mortezaei F., Royan M., Allaf Noveirian H., Babakhani1 A., Alaie Kordghashlaghi H., Balcazar J.L. (2020). In vitro assessment of potential probiotic characteristics of indigenous *Lactococcus lactis* and *Weissella oryzae* isolates from rainbow trout (*Oncorhynchus mykiss* Walbaum). J. Appl. Microbiol..

[66] Daza C., Campos V., Rojas C., Rodríguez-Llamazares S., Smith C., Mondaca M. (2016). Reduction of selenite to elemental Selenium by *Pantoea agglomerans*. Gayana.

[67] Oremland R.S., Herbel M.J., Switzer-Blum J., Langley S., Beveridge T.J., Ajayan P.M., Sutto T., Ellis A.V., Curran S. (2004). Structural and spectral features of selenium nanospheres produced by Se-respiring bacteria. Appl. Environ. Microbiol..

[68] Dhanjal S., Cameotra S.S. (2010). Aerobic biogenesis of selenium nanospheres by *Bacillus cereus* isolated from coalmine soil. Microb. Cell Factories.

[69] Torres S.K., Campos V.L., León C.G., Rodríguez-Llamazares S.M., Rojas S.M., González M., Smith C.T., Mondaca M.A. (2012). Biosynthesis of selenium nanoparticles by *Pantoea agglomerans* and their antioxidant activity. J. Nanoparticle Res..

[70] Wang Q., Garrity G.M., Tiedje J.M., Cole J.R. (2007). Naive Bayesian Classifier for Rapid Assignment of rRNA Sequences into the New Bacterial Taxonomy. Appl. Environ. Microbiol..

[71] Campos V.L., León C., Mondaca M.A., Yáñez J., Zaror C. (2011). Arsenic mobilization by epilithic bacterial communities associated with volcanic rocks from Camarones River Atacama Desert Northern Chile. Arch. Environ. Contam. Toxicol..

[72] Valenzuela A., Campos V., Yañez F., Alveal K., Gutiérrez P., Rivas M., Contreras N., Klempau A., Fernandez I., Oyarzun C. (2012). Application of artificial photoperiod in fish: A factor that increases susceptibility to infectious diseases?. Fish Physiol. Biochem..

[73] Vera B. (2016). Bio-Obtención de Nanopartículas de Selenio y su Potencial Aplicación Como Suplemento Alimentario Inmunoestimulante en Trucha Arcoíris (*Oncorhynchus mykiss*). Bachelor’s Thesis.

[74] Brown L.A., Stoskopf M.K. (1993). Anaesthesia and restraint. Fish Medicine, W.B..

[75] Coyle S.D., Durborow R.M., Tidwell J.H. (2004). Anesthetics in aquaculture. South. Reg. Aquac. Cent. SRAC.

[76] Hu Y., Maisey K., Subramani A.A., Liu F., Flores-Kossack C., Imarai M., Secombes C.J., Wang T. (2018). Characterisation of rainbow trout peripheral blood leucocytes prepared by hypotonic lysis of erythrocytes, and analysis of their phagocytic activity, proliferation and response to PAMPs and proinflammatory cytokines. Dev. Comp. Immunol..

[77] Anderson D., Siwicki A., Stolen J., Anderson D., Zelikoff S., Twerdok L., Kaattari S. (1993). Measuring the effects of contaminants on fish by haematological and serological methods. Modulators of Fish Immune Responses.

[78] Takemura A., Takano K. (1995). Lysozyme in the ovary of tilapia (*Oreochromis mossambicus*): Its purification and some biological properties. Fish Physiol. Biochem..

[79] Lawrence R., Burk R. (1976). Glutathione peroxidase activity in selenium-deficient rat live. Biochem. Biophys. Res. Commun..

[80] Fontagne’-Dicharry S., Godin S., Liu H., Prabhu P.A.J., Bouyssière B., Bueno M., Tacon P., Médale F., Kaushik S. (2015). Influence of the forms and levels of dietary selenium on antioxidant status and oxidative stress-related parameters in rainbow trout (*Oncorhynchus mykiss*) fry. Br. J. Nutr..

[81] Lowry O.H., Rosebrough N.J., Farr A.L., Randall R.J. (1951). Protein Measurement with the Folin Phenol Reagent. J. Biol. Chem..

[82] Naderi M., Keyvanshokooh S., Salati A.P., Ghaedi A. (2017). Proteomic analysis of liver tissue from rainbow trout (*Oncorhynchus mykiss*) under high rearing density after administration of dietary vitamin E and selenium nanoparticles. Comp. Biochem. Physiol. Part D Genom. Proteom..

[83] Lugert V., Thaller G., Tetens J., Schulz C., Krieter J. (2014). A review on fish growth calculation: Multiple functions in fish production and their specific application. Rev. Aquac..

[84] Denev S., Staykov Y., Moutafchieva R., Beev G. (2009). Microbial ecology of the gastrointestinal tract of fish and the potential application of probiotics and prebiotics in finfish aquaculture. Int. Aquat. Res..

[85] Hai N.V. (2015). The use of probiotics in aquaculture. J. Appl. Microbiol..

[86] Dawood M.A.O., Koshio S., Abdel-Daim M.M., Van Doan H. (2019). Probiotic application for sustainable aquaculture. Rev. Aquac..

[87] Valipour A., Nadaei S., Noori A., Khanipour A.A., Hoseinifar S.H. (2019). Dietary *Lactobacillus plantarum* affected on some immune parameters, air-exposure stress response, intestinal microbiota, digestive enzyme activity and performance of narrow clawed crayfish (*Astacus leptodactylus*, Eschscholtz). Aquaculture.

[88] Zhai Q., Wang H., Tian F., Zhao J., Zhang H., Chen W. (2017). Dietary *Lactobacillus plantarum* supplementation decreases tissue lead accumulation and alleviates lead toxicity in Nile tilapia (*Oreochromis niloticus*). Aquac. Res..

[89] Silarudee S., Tongpim S., Charoensri N., Doolgindachbaporn S. (2019). Effect of a Probiotic *Lactobacillus plantarum* CR1T5 Dietary Supplements on Non-specific Immunity in Black Eared Catfish (*Pangasius larnaudii*). J. Pure Appl. Microbiol..

[90] Shakibaie M., Mohammadi-Khorsand T., Adeli-Sardou M., Jafari M., Amirpour-Rostami S., Ameri A., Forootanfar H. (2017). Probiotic and antioxidant properties of selenium-enriched *Lactobacillus brevis* LSe isolated from an Iranian traditional dairy product. J. Trace Elem. Med. Biol.

[91] Kang S., Li R., Jin H., You H.J., Ji G.E. (2020). Effects of Selenium- and Zinc-Enriched *Lactobacillus plantarum* SeZi on Antioxidant Capacities and Gut Microbiome in an ICR Mouse Model. Antioxidants.

[92] Shu G., Mei S., Chen L., Zhang B., Guo M., Cui X., Chen H. (2020). Screening, identification, and application of selenium-enriched *Lactobacillus* in goat milk powder and tablet. J. Food Process. Preserv..

[93] Fečkaninová A., Koščová J., Mudroňová D., Schusterová P., Cingeľová I., Maruščáková C., Popelka P. (2019). Characterization of two novel lactic acid bacteria isolated from the intestine of rainbow trout (*Oncorhynchus mykiss*, Walbaum) in Slovakia. Aquaculture.

[94] Savadogo A., Ouattara C., Bassole I., Traore A. (2004). Antimicrobial Activities of Lactic Acid Bacteria Strains Isolated from Burkina Faso Fermented Milk. Pak. J. Nutr..

[95] Tebyanian H., Bakhtiari A., Karami A., Kariminik A. (2017). Antimicrobial Activity of some *Lactobacillus* Species against Intestinal Pathogenic Bacteria. Int. Lett. Nat. Sci..

[96] Saulnier D.M., Spinler J.K., Gibson G.R., Versalovic J. (2009). Mechanisms of probiosis and prebiosis: Considerations for enhanced functional foods. Curr. Opin. Biotechnol..

[97] Shokryazdan P., Sieo C., Kalavathy R., Liang J., Alitheen N., Jahromi M., Ho Y. (2014). Probiotic Potential of *Lactobacillus* Strains with Antimicrobial Activity against Some Human Pathogenic Strains. BioMed Res. Int..

[98] European Food Safety Authority (EFSA) (2012). Panel on Additives and Products or Substances used in Animal Feed (FEEDAP). Guidance on the assessment of bacterial susceptibility to antimicrobials of human and veterinary importance. EFSA J..

[99] Bujnakova D., Strakova E. (2017). Safety, probiotic and technological properties of *Lactobacilli* isolated from unpasteurised ovine and caprine cheeses. Ann. Microbiol..

[100] Rowe G.E., Welch R.A. (1994). Assays of hemolytic toxins. Methods Enzym..

[101] Ramachandran G. (2013). Gram-positive and Gram-negative bacterial toxins in sepsis: A brief review. Virulence.

[102] Yasmin I., Saeed M., Khan W.A., Khaliq A., Chughtai M.F.J., Iqbal R., Tehseen S., Naz S., Liaqat A., Mehmood T. (2020). In Vitro Probiotic Potential and Safety Evaluation (Hemolytic, Cytotoxic Activity) of *Bifidobacterium* Strains Isolated from Raw Camel Milk. Microorganisms.

[103] Sharma K., Sharma N., Sharma R. (2016). Identification and evaluation of in vitro probiotic attributes of novel and potential strains of lactic acid bacteria isolated from traditional dairy products of North-West Himalayas. J. Clin. Microbiol. Biochem. Technol..

[104] Tokatlı M., Gülgör G., Bağder Elmaci S., Arslankoz İşleyen N., Özçelik F. (2015). In vitro properties of potential probiotic indigenous lactic acid bacteria originating from traditional pickles. BioMed Res. Int..

[105] Sica M.G., Brugnoni L.I., Marucci P.L., Cubitto M.A. (2012). Characterization of probiotic properties of lactic acid bacteria isolated from an estuarine environment for application in rainbow trout (*Oncorhynchus mykiss*, Walbaum) farming. Antonie Van Leeuwenhoek.

[106] Bravo J.P., Hernandez A.J., Morales G., Dantagnan P., Márquez L. (2018). Digestive coordination of the gastric function in Atlantic salmon *Salmo salar* juveniles. Lat. Am. J. Aquat. Res..

[107] Lückstädt C. (2008). The use of acidifiers in fish nutrition. CAB Rev. Perspect. Agric. Vet..

[108] Fečkaninová A., Koščová J., Mudroňová D., Popelka P., Toropilová J. (2017). The use of probiotic bacteria against *Aeromonas* infections in salmonid aquaculture. Aquaculture.

[109] Iorizzo M., Albanese G., Letizia F., Testa B., Tremonte P., Vergalito F., Lombardi S.J., Succi M., Coppola R., Sorrentino E. (2022). Probiotic Potentiality from Versatile *Lactiplantibacillus plantarum* strains as resource to enhance freshwater fish health. Microorganisms.

[110] Fontana L., Brito M.B., Diaz J.P., Quezada S.M., Gil A. (2013). Sources, isolation, characterization and evaluation of probiotics. Br. J. Nutr..

[111] Begley M., Hill C., Gahan C.G. (2006). Bile salt hydrolase activity in probiotics. Appl. Environ. Microbiol..

[112] Martínez F.G., Moreno-Martin G., Pescuma M., Madrid-Albarrán Y., Mozzi F. (2020). Biotransformation of Selenium by Lactic Acid Bacteria: Formation of Seleno-Nanoparticles and Seleno-Amino. Front. Bioeng. Biotechnol..

[113] Ogasawara Y., Lacourciere G.M., Ishii K., Stadtman T.C. (2005). Characterization of potential selenium-binding proteins in the selenophosphate synthetase system. Proc. Natl. Acad. Sci. USA.

[114] Ravanal J. (2015). Determinación de Proteínas que Participan en el Control del Tamaño de Nanopartículas de Selenio Elemental Producidas por Pantoea Agglomerans. Bachelor’s Thesis.

[115] Xia S.K., Chen L., Liang J.Q. (2007). Enriched selenium and its effects on growth and biochemical composition in *Lactobacillus bulgaricus*. J. Agric. Food Chem..

[116] Eszenyi P., Sztrik A., Babka B., Prokisch J. (2011). Elemental, Nano-sized (100–500 nm) selenium production by probiotic lactic acid bacteria. Int. J. Biosci. Biochem. Bioinform..

[117] Zhang W., Chen Z., Liu H., Zhang L., Gao P., Li D. (2011). Biosynthesis and structural characteristics of selenium nanoparticles by *Pseudomonas alcaliphila*. Colloids Surf. B Biointerfaces.

[118] Hosnedlova B., Kepinska M., Skalickova S., Fernandez C., Ruttkay-Nedecky B., Peng Q., Baron M., Melcova M., Opatrilova R., Zidkova J. (2018). Nano-selenium and its nanomedicine applications: A critical review. Int. J. Nanomed..

[119] Daming R., Yingyu W., Zilai W., Jun C., Hekui L., Jingye Z. (2003). Complete DNA sequence and analysis of two cryptic plasmids isolated from *Lactobacillus plantarum*. Plasmid.

[120] Enferadi M.H.N., Mohammadizadeh F., Soltani M., Bahri A.H., Sheikhzadeh N. (2018). Effects of *Lactobacillus plantarum* on Growth Performance, Proteolytic Enzymes Activity and Intestine Morphology in Rainbow Trout (*Oncorhynchus mykiss*). Turk. J. Fish. Aquat. Sci..

[121] Medina M., Sotil G., Flores V., Fernández C., Sandoval N. (2020). In vitro assessment of some probiotic properties and inhibitory activity against *Yersinia ruckeri* of bacteria isolated from rainbow trout *Oncorhynchus mykiss* (Walbaum). Aquac. Rep..

[122] Uribe-Querol E., Rosales C. (2020). Phagocytosis: Our Current Understanding of a Universal Biological Process. Front. Immunol..

[123] Dalmo R.A., Ingebrigtsen K., Bøgwald J. (1997). Non-specific defence mechanisms in fish, with particular reference to the reticuloendothelial system (RES). J. Fish Dis..

[124] Rodríguez A., Esteban M.A., Meseguer J. (2003). Phagocytosis and peroxidase release by seabream (*Sparus aurata* L.) leucocytes in response to yeast cells. Anat. Rec. A Discov. Mol. Cell. Evol. Biol..

[125] Soltani M., Abdy E., Alishahi M., Taheri Mirghaed A., Hosseini-Shekarabi P. (2017). Growth performance, immune-physiological variables and disease resistance of common carp (*Cyprinus carpio*) orally subjected to different concentrations of *Lactobacillus plantarum*. Aquacult. Int..

[126] Chen L., Pan D.-D., Zhou J., Jiang Y.-Z. (2005). Protective effect of selenium-enriched lactobacillus on CCl4-induced liver injury in mice and its possible mechanisms. World J. Gastroenterol..

[127] Saurabh S., Sahoo P.K. (2008). Lysozyme: An important defence molecule of fish innate immune system. Aquac. Res..

[128] Fast M.D., Simsa D.E., Burka J.F., Mustafa A., Ross N.W. (2002). Skin morphology and humoral non-specific defence parameters of mucus and plasma in rainbow trout, coho and *Atlantic salmon*. Comp. Biochem. Physiol. Part A Mol. Integr. Physiol..

[129] Kane A.M., Soltani M., Ebrahimzahe-Mousavi H., Pakzad K. (2016). Influence of probiotic, *Lactobacillus plantarum* on serum biochemical and immune parameters in vaccinated rainbow trout (*Oncorhynchus mykiss*) against streptococcosis/lactococosis. Int. J. Aquat. Biol..

[130] Hang B.T., Balami S., Phuong N.T. (2022). Effect of *Lactobacillus plantarum* on growth performance, immune responses, and disease resistance of striped catfish (*Pangasianodon hypophthalmus*). AACL Bioflux.

[131] Son V.M., Chang C.-C., Wu M.-C., Guu Y.-K., Chiu C.-H., Cheng W. (2009). Dietary administration of the probiotic, *Lactobacillus plantarum*, enhanced the growth, innate immune responses, and disease resistance of the grouper *Epinephelus coioides*. Fish Shellfish Immunol..

[132] Yanez-Lemus F., Moraga R., Mercado L., Jara-Gutierrez C., Smith C., Aguayo P., Sanchez-Alonzo K., García-Cancino A., Valenzuela A., Campos L. (2022). Selenium nanoparticles biosynthesized by *Pantoea agglomerans* and their effects on cellular and physiological parameters in the rainbow trout *Oncorhynchus mykiss*. Biology.

[133] Le K.T., Fotedar R. (2014). Bioavailability of selenium from different dietary sources in yellowtail kingfish (*Seriola lalandi*). Aquaculture.

[134] Dawood M.A.O., Zommara M., Eweedah N.M., Helal A.I., Aboel-Darag M.A. (2020). The potential role of nano-selenium and vitamin C on the performances of Nile tilapia (*Oreochromis niloticus*). Environ. Sci. Pollut. Res..

[135] Shang X., Wang B., Sun Q., Zhang Y., Lu Y., Liu S., Li Y. (2022). Selenium-enriched *Bacillus subtilis* reduces the effects of mercury-induced on inflammation and intestinal microbes in carp (*Cyprinus carpio* var. specularis). Fish Physiol. Biochem..

[136] Lushchak V.I., Bagnyukova T.V. (2006). Effects of different environmental oxygen levels on free radical processes in fish. Comp. Biochem. Physiol. Part B Biochem. Mol. Biol..

[137] Magnoni L., Novais S.C., Silva C.O., Lemos M.F., Ozorio R., Geurden I., Leguen I., Prunet P., Eding E., Schrama J. The impact of nutritional and environmental stressors on the immune response, oxidative stress and energy use of rainbow trout (*Oncorhynchus mykiss*). Proceedings of the International Meeting on Marine Research 2016.

[138] Mengistu B.M., Bitsue H.K., Huang K. (2021). The Effects of Selenium-Enriched Probiotics on Growth Performance, Oocysts Shedding, Intestinal Cecal Lesion Scores, Antioxidant Capacity, and mRNA Gene Expression in Chickens Infected with *Eimeria tenella*. Biol. Trace Elem. Res..

[139] Tollerz Bratteby U. (2019). Factors Explaining Variation in the Fecundity of Female Baltic Salmon (*Salmo salar*)–The Influence of Length, Body Condition and Growth Rate at Sea. Master’s Thesis.

[140] Melo-Bolívar J., Ruiz R., Hume M., Villamil L., Alzate A., Cañas B., Pérez-Munguia S., Hernandez-Mendoza H., Pérez-Conde C., Gutiérrez A.M. (2007). Evaluation of the Inorganic Selenium Biotransformation in Selenium-Enriched Yogurt by HPLC-ICP-MS. J. Agric. Food Chem..

[141] Van Zyl W., Deane S., Dicks L. (2020). Molecular insights into probiotic mechanisms of action employed against intestinal pathogenic bacteria. Gut Microbes.

[142] Ringø E. (2020). Probiotics in shellfish aquaculture. Aquac. Fish..

[143] Van Doan H., Hoseinifar S.H., Ringø E., Esteban M.A., Dadar M., Dawood M.A.O., Faggio C. (2019). Host-Associated Probiotics: A Key Factor in Sustainable Aquaculture. Rev. Fish. Sci. Aquac..

[144] Jatobá A., Pereira M.O., Vieira L.M., Bitencourt M., Rodrigues E., Fachini F.A., Moraes A.V. (2018). Action time and feed frequency of *Lactobacillus plantarum* for Nile tilapia. Arq. Bras. Med. Vet. Zootec..

[145] Parthasarathy R., Ravi D. (2011). Probiotic bacteria as growth promoter and biocontrol agent against *Aeromonas hydrophila* in *Catla catla* (Hamilton, 1822). Indian J. Fish..

[146] Giri S.S., Sukumaran V., Oviya M. (2013). Potential probiotic *Lactobacillus plantarum* VSG3 improves the growth, immunity, and disease resistance of tropical freshwater fish, *Labeo rohita*. Fish Shellfish Immunol..

[147] El-Kader M.F.A., El-Bab A.F.F., Abd-Elghany M.F., Abdel-Warith A.-W.A., Younis E.M., Dawood M.A.O. (2021). Selenium Nanoparticles Act Potentially on the Growth Performance, Hemato-Biochemical Indices, Antioxidative, and Immune-Related Genes of European Seabass (*Dicentrarchus labrax*). Biol. Trace Elem. Res..

[148] Zhou X., Wang Y., Gu Q., Li W. (2009). Effects of different dietary selenium sources (selenium nanoparticle and selenomethionine) on growth performance, muscle composition and glutathione peroxidase enzyme activity of crucian carp (*Carassius auratus gibelio*). Aquaculture.

[149] Yang L., Wang J., Huang K., Liu Q., Liu G., Xu X., Zhang H., Zhu M. (2021). Selenium-enriched *Bacillus subtilis* yb-114246 improved growth and immunity of broiler chickens through modified ileal bacterial composition. Sci. Rep..

